# Exploring the Role of *Pseudomonas aeruginosa* Elastase in Lung Epithelial Barrier Dysfunction: Advancing toward Antivirulence Therapies

**DOI:** 10.1021/acsinfecdis.5c00915

**Published:** 2026-04-10

**Authors:** Roya Shafiei, Alaa Alhayek, Lukas Hiller, Lorenz Latta, Tobias Neu, Ahmad Aljohmani, Sahar Abdollahibiroun, Eva-Maria Schönborn, Daniela Yildiz, Nicole Schneider-Daum, Claus-Michael Lehr, Jörg Haupenthal, Anna K. H. Hirsch

**Affiliations:** † Helmholtz-Institute for Pharmaceutical Research Saarland (HIPS), Campus E8.1, 66123 Saarbrücken, Germany; ‡ 443745 Helmholtz Centre for Infection Research (HZI), Inhoffenstraße 7, 38124 Braunschweig, Germany; § Department of Pharmacy, Saarland University, 66123 Saarbrücken, Germany; ∥ Institute of Experimental and Clinical Pharmacology and Toxicology, PZMS, ZHMB, Saarland University, Kirrbergerstraße 100, 66421 Homburg/Saar, Germany; ⊥ PharmaScienceHub (PSH), Campus A2.3, 66123 Saarbrücken, Germany; # German Center for Infection Research (DZIF), Inhoffenstraße 7, 38124 Braunschweig, Germany

**Keywords:** *Pseudomonas aeruginosa*, LasB, antivirulence therapies, tight junctions, *DUSP2*, *FGFBP1*

## Abstract

*Pseudomonas aeruginosa*, a major cause of pulmonary infections, poses significant clinical challenges due to its virulence and rising antimicrobial resistance. We investigated the role of LasB, a key virulence factor and elastase of *P. aeruginosa*, in disrupting the lung epithelial barrier. LasB cleaves the junctional protein E-cadherin, alters Claudin-4 localization, and reduces levels of immunomodulatory cytokines including GM-CSF and G-CSF. Using bronchial and alveolar cell models, we demonstrate that LasB induces dose-dependent barrier damage in both systems. Transcriptomic analysis reveals widespread gene expression changes, including the upregulation of *DUSP2* and *FGFBP1* associated with stress signaling and immune modulation. LasB inhibitors mitigate barrier disruption and partially restore cytokine levels. In a live bacterial infection model, LasB inhibition supports antibiotic treatment, enhancing bacterial clearance and preserving epithelial integrity. These findings establish LasB as a pivotal factor in *P. aeruginosa* pathogenesis and highlight the therapeutic potential of antivirulence strategies targeting LasB as promising adjuncts to conventional antibiotics.

## Graphical abstract

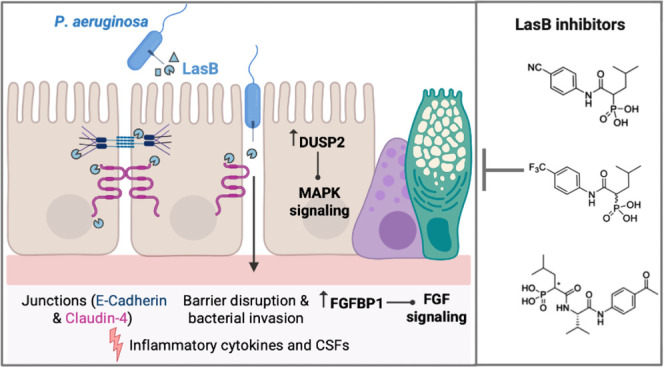

## 1 Introduction

The escalating problem of antimicrobial resistance (AMR) is one of the greatest global health challenges, with *Pseudomonas aeruginosa* emerging as a major cause of antibiotic-resistant pulmonary infections.^1^ This opportunistic pathogen poses a formidable threat due to its intrinsic virulence, diverse adaptation strategies, and ability to cause severe infections and high mortality even in the absence of AMR.^2^ Traditional antibiotics targeting bacterial viability are becoming increasingly ineffective against *P. aeruginosa*.^3^ The ongoing rise in bacterial resistance, biofilm formation, and immune evasion mechanisms reduce antibiotic efficacy, leading to prolonged hospital stays, increased healthcare costs, and higher mortality rates.^4,5^ Consequently, there is an urgent need for resistance-agnostic therapeutic strategies that exert minimal selection pressure. Antivirulence strategies offer a promising alternative by disrupting bacterial pathogenic mechanisms rather than targeting viability, thereby reducing the risk of resistance while preserving the host-beneficial microbiota.^6^

*P. aeruginosa* elastase (LasB) — secreted via the type II secretion system — plays a central role in pathogenicity.^7^ This extracellular metalloprotease degrades host structural proteins such as elastin, collagen, and laminin, thereby facilitating tissue invasion and bacterial survival.^8,9^ LasB also targets key immune components such as human immunoglobulins A and G, as well as pro-inflammatory cytokines like TNF, IL-1β, IFN-γ, and IL-6, exacerbating the infection severity.^8,10,11^ Although the role of LasB in pathogenesis is well-studied, its precise mechanism in lung infections–as well as how its inhibition might preserve the lung barrier and enhance antibiotic efficacy–remains poorly understood.

The lung epithelial barrier is crucial for protection against pathogens and environmental stressors.^12^ Bronchial and alveolar epithelial cells — represented by the human lung adenocarcinoma cell line Calu-3 and the monoclonal human alveolar epithelial cell line Arlo, respectively — serve as physiologically relevant in vitro models for studying lung infections.^13,14^ Calu-3 cells mimic key features of the bronchial epithelium in vivo, forming polarized monolayers with tight junctions — e.g., Zonula Occludens-1 (ZO-1) and E-cadherin — and exhibiting transepithelial electrical resistance (TEER) values reaching ∼300 Ω·cm^2^ under air–liquid interface (ALI) culture conditions.^15,16^ In contrast, Arlo cells model the alveolar epithelium with high TEER values (up to ∼3000 Ω·cm^2^ under ALI conditions), providing improved barrier properties and closely mimicking the alveolar epithelium in vivo.^17^ Importantly, Arlo cells express genes relevant to barrier integrity and homeostasis, similar to primary human alveolar epithelial cells (hAEpC), offering a more physiologically relevant model for human-specific studies.^17^ Models based on human cells and tissues are promising alternatives to animal experiments, allowing for the isolation of essential human biological processes and making them accessible in vitro.^17,18^ Together, these models of bronchial and alveolar epithelial cells provide a platform for investigating the impact of LasB on epithelial integrity and immune modulation.

This study aims to elucidate the biological impact of LasB on lung epithelium, focusing on its cellular and molecular mechanisms of action. LasB disrupts junctional proteins like occludin and VE-cadherin, leading to increased permeability and barrier dysfunction.^8^ This effect is observed not only in lung models but also in other epithelial systems such as the corneal epithelium, and in mouse models where LasB induces severe lung injury and diffuse alveolar damage.^19,20^ While LasB and antivirulence agents have been well studied in recent years, physiologically relevant in vitro studies on lung cells that reflect the in vivo conditions remain limited.^21–24^ In particular, the role of LasB in immune modulation at the transcriptomic level remains to be investigated. Our research employs filter-based models in liquid-covered conditions (LCC) and transcriptomic analyses to investigate the effects of LasB on epithelial barriers, immune responses, and gene expression. This approach enables highly reproducible, multiparametric barrier and imaging readouts under controlled experimental conditions.

Our findings reveal a complex interplay of cellular responses, with LasB inducing diverse changes in gene expression patterns, particularly in pathways related to bacterial infection, cellular processes, and immune modulation, including FGF and MAPK signaling. These insights deepen our understanding of the molecular mechanisms underlying *P. aeruginosa* pathogenesis and highlight critical targets for therapeutic intervention. The identification of consistent gene expression patterns across experimental conditions advances diagnostic strategies by uncovering biomarkers to track disease progression and assess treatment efficacy, addressing a significant gap in evaluating antivirulence therapies in vivo and in clinical settings.^25,26^ By integrating functional assays, protein analyses, and transcriptomic studies, this study lays the groundwork for developing targeted therapies that synergize with antibiotics or immune modulators, offering a dual strategy to enhance bacterial clearance and preserve epithelial integrity in clinical settings.

## 2 Results and Discussion

### 2.1 LasB-Mediated Disruption of Epithelial Integrity and Immune Modulation in Bronchial and Alveolar Cells

Maintaining epithelial barrier integrity is critical for preventing microbial invasion and preserving lung function. In *P. aeruginosa* infections, the virulence factor LasB plays a key role in compromising this barrier by targeting junctional proteins, thereby undermining host defenses.^20,27^ To assess the impact of LasB on lung epithelial integrity, we examined LasB-mediated changes in transepithelial electrical resistance (TEER), paracellular permeability to fluorescein sodium (FluNa), immune responses, and cleavage of the tight junction protein E-cadherin in bronchial Calu-3 and alveolar Arlo cells (Figure 1A). Together, these readouts captured barrier dynamics in models closely resembling in vivo lung conditions. TEER and FluNa permeability are inversely related metrics of epithelial integrity, where higher TEER reflects a more intact barrier, while increased FluNa permeability reflects junctional disruption.^28^

**Figure 1:**
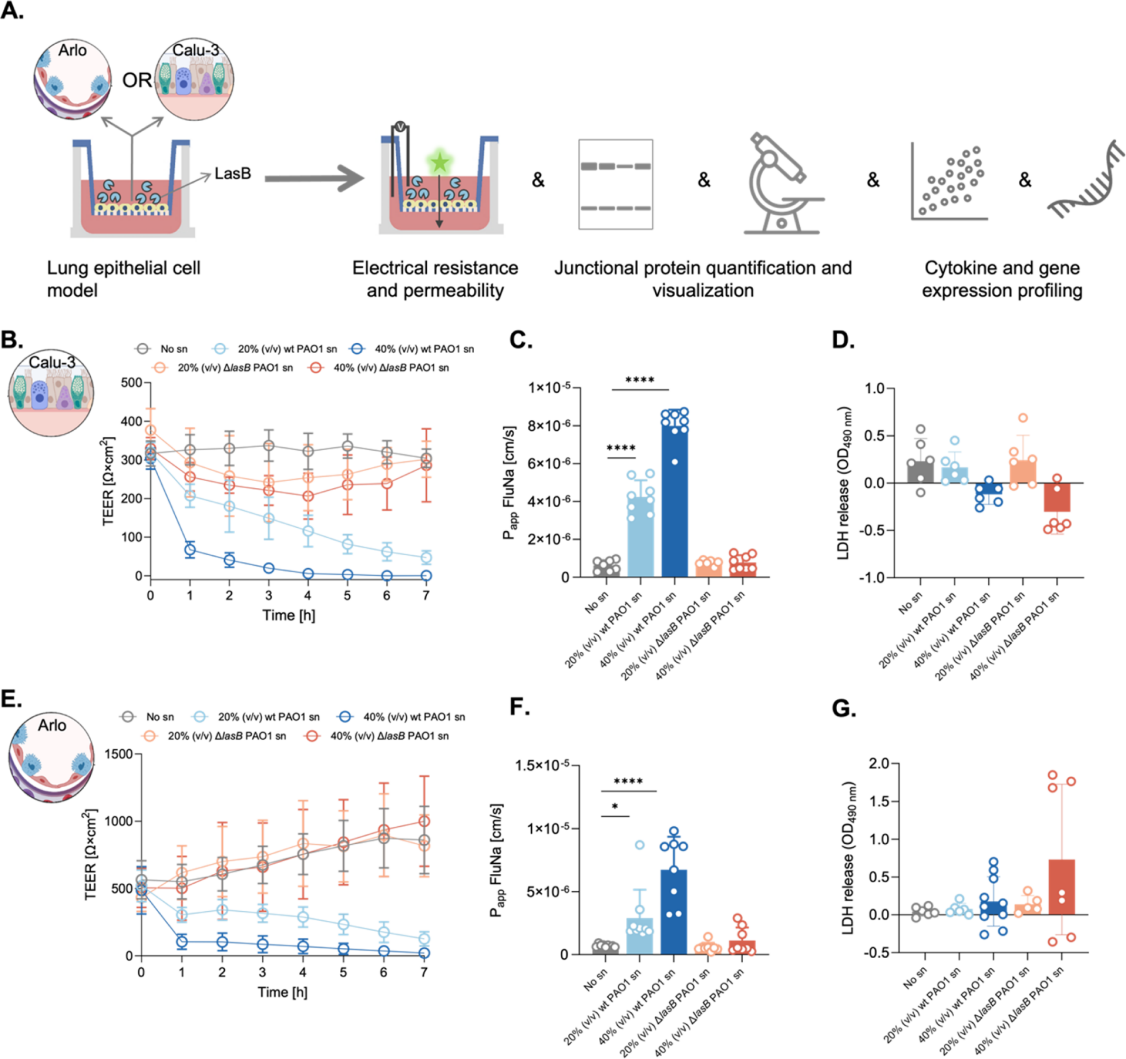
Experimental workflow and epithelial barrier disruption in Calu-3 and Arlo cells. (A) Overview of the experimental workflow. Calu-3 and Arlo cells were cultured on Transwell inserts until stable transepithelial electrical resistance (TEER) was achieved, followed by treatment with 20% and 40% (v/v) supernatants (sn) from *Pseudomonas aeruginosa* PAO1 wild-type (wt) and *lasB* knockout (Δ*lasB*) strains. Downstream analyses included TEER measurement, permeability of fluorescein sodium (FluNa), lactate dehydrogenase (LDH) release, junctional protein analysis, cytokine profiling, and gene expression. (B–D) Barrier function in Calu-3 cells: (B) TEER, (C) FluNa permeability (P_app_), and (D) LDH release under the indicated conditions. (E–G) Corresponding analyses in Arlo cells: (E) TEER, (F) FluNa permeability, and (G) LDH release. Data represent mean ± standard deviation (*n* = 3 independent experiments, each sample with 2 replicates within an experiment). Statistical analyses were performed using ordinary one-way ANOVA followed by Dunnett’s multiple comparisons test comparing the mean value of each group with wt PAO1 sn (****p ≤ 0.0001; ***p ≤ 0.001; *p ≤ 0.05).

#### 2.1.1 LasB Effect on Calu-3 Cells: Barrier Disruption

In Calu-3 cells, exposure to bacterial supernatant (sn) from wild-type (wt) LasB-producing *P. aeruginosa* PAO1 induced a concentration-dependent decrease in TEER, reaching 77.9% reduction within 1 h of exposure to 40 wt % PAO1 sn and near-complete barrier loss by 7 h (Figure 1B). This decline was accompanied by a significant increase in FluNa permeability, confirming the compromised barrier function (Figure 1C). In contrast, sn from the LasB-deficient PAO1 mutant (Δ*lasB*) caused only moderate TEER reductions and minimal increases in FluNa permeability, with signs of partial recovery over time. This suggests that the absence of LasB mitigates junctional damage. Furthermore, clinical isolates NH57388A muc. and RP73, which express low levels of *lasB* (Figure S1) induced only limited changes in TEER and FluNa permeability, further supporting the central role of LasB in epithelial barrier disruption (Figure S2).^29^

To determine the quantity of LasB required to disrupt the epithelial barrier, we treated Calu-3 cells with increasing concentrations of purified LasB. TEER measurements showed a clear, dose-dependent barrier-disrupting effect (Figure S3), with initial signs of disruption already evident at concentrations as low as 3 nM. Notably, the barrier disruption caused by 20 and 40 wt % PAO1 sn was comparable to that induced by approximately 100 nM and 200 nM of purified LasB, respectively. These findings underscore the clinical challenge that even minimal LasB levels strongly compromise barrier integrity.

#### 2.1.2 LasB Effect on Arlo Cells: Differential Resistance

Despite their inherently tighter barriers, Arlo cells also exhibited a concentration-dependent decline in TEER upon exposure to wt PAO1 sn, with reductions of up to 95.7% after 40% sn treatment relative to untreated (no sn) controls (Figure 1E). However, the extent of barrier disruption was less pronounced than in Calu-3 cells, as reflected by a smaller increase in FluNa permeability (Figure 1F). This indicates that the inherently stronger barrier properties of Arlo cells provide partial resistance to LasB-mediated damage. In contrast, exposure to Δ*lasB* PAO1 sn resulted in sustained TEER and minimal changes in FluNa permeability, suggesting that the absence of LasB preserves barrier function and may activate recovery mechanisms.

To confirm the noninvasive nature of this assay, we measured lactate dehydrogenase (LDH) release during the 7 h exposure period. No significant differences in LDH levels were observed between treated and control groups in both cell types, indicating that LasB-mediated barrier disruption occurs independently of substantial cell death (Figure 1D,G). These results demonstrate that LasB damages epithelial barriers at low, noncytotoxic concentrations, highlighting its ability to impair integrity without killing cells.

#### 2.1.3 Molecular Mechanisms of LasB-Mediated Barrier Disruption

To elucidate the molecular mechanisms underlying LasB-induced epithelial barrier disruption, we investigated the cleavage of the tight junction protein E-cadherin, a critical mediator of epithelial integrity.^30^ We observed complex, cell-type-specific responses in E-cadherin dynamics following LasB exposure.

In Calu-3 cells, Western blot analysis showed significant cleavage of E-cadherin upon exposure to wt PAO1 sn compared to Δ*lasB* PAO1 sn and no sn control (Figure 2A,B). Interestingly, confocal laser scanning microscopy (CLSM) revealed a strong E-cadherin signal in wt PAO1 sn-treated Calu-3 cells versus weaker signals in control conditions (Figure 2C). This apparent discrepancy, coupled with the absence of significant changes in E-cadherin gene expression (Figure S4), suggests a post-translational response, possibly involving redistribution of existing E-cadherin to the cell surface, reduced protein turnover, or activation of a preexisting pool of E-cadherin as a compensatory response to junctional disruption. Notably, different antibodies were employed for Western blot and CLSM due to compatibility constraints. Thus, partial cleavage of E-cadherin may abolish detection by Western blot, while the CLSM antibody could still recognize intact epitopes, potentially accounting for the observed discrepancy.

**Figure 2:**
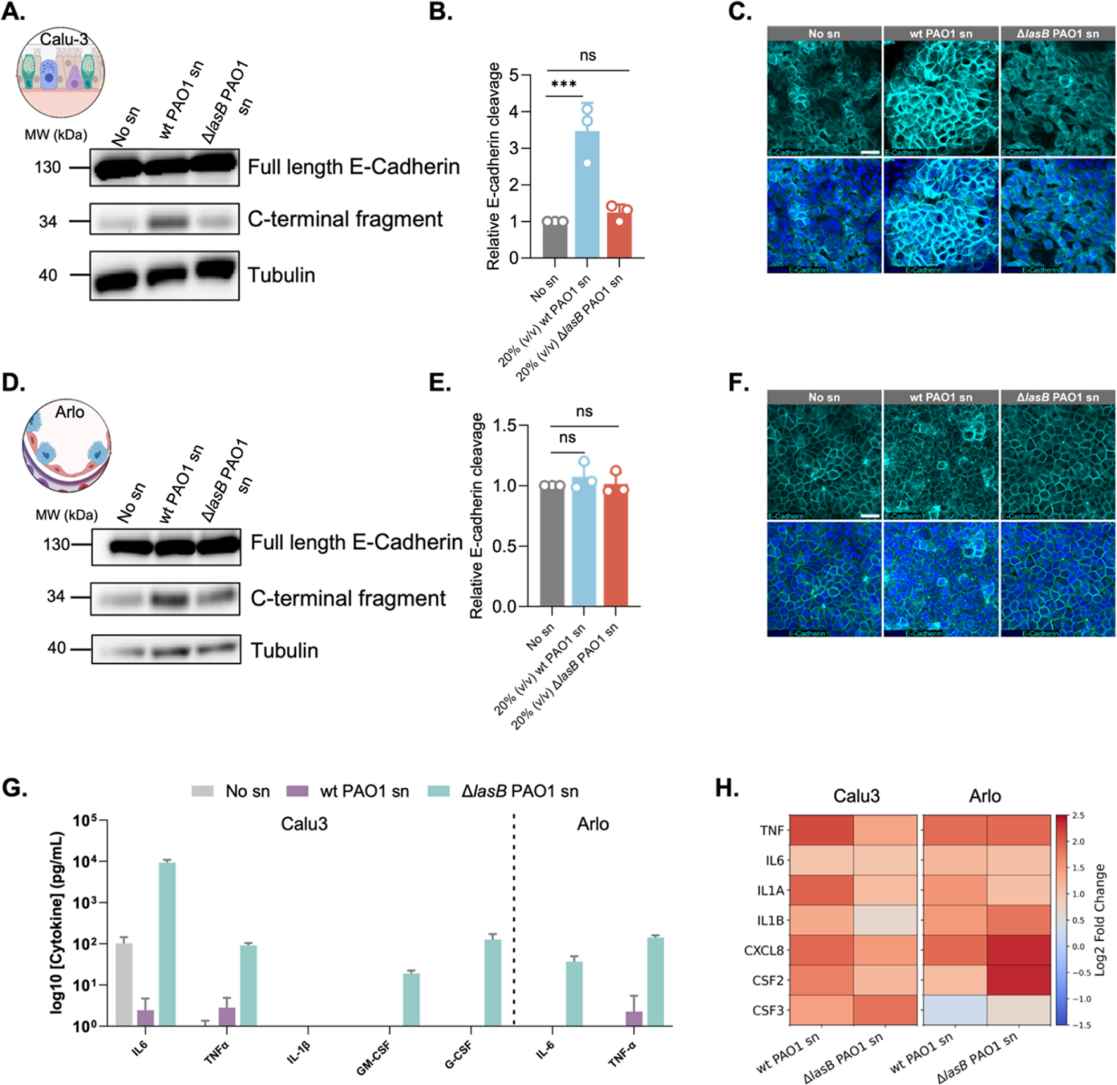
LasB-mediated cleavage of E-cadherin and modulation of inflammatory responses in bronchial and alveolar epithelial cells. (A,D) Western blot analysis of E-cadherin and C-terminal fragments in (A) Calu-3 and (D) Arlo cells treated with 20% (v/v) *Pseudomonas aeruginosa* wild-type (wt) PAO1 supernatant (sn), Δ*lasB* PAO1 sn, or no sn (control). (B,E) Quantification of E-cadherin cleavage relative to total E-cadherin levels. Data represent mean ± SD (*n* = 3). Statistical analyses were performed using one-way ANOVA with Dunnett’s multiple comparisons test (***p ≤ 0.001; ns, not significant). (C,F) Confocal microscopy of E-cadherin (cyan) and DAPI (blue) in (C) Calu-3 and (F) Arlo cells. Scale bars: 25 μm. (G) Cytometric bead array (CBA) quantification of secreted inflammatory cytokines and colony-stimulating factors (CSFs) in bronchial Calu-3 (left) and alveolar Arlo (right) cells under healthy conditions, or following challenge with wt PAO1 sn or Δ*lasB* PAO1 sn. Data are presented as log_10_-transformed concentrations (pg/mL). (H) Gene expression profiles of key inflammatory mediators in bronchial Calu-3 (left) and alveolar Arlo (right) cells following exposure to wt PAO1 sn or Δ*lasB* PAO1 sn, displayed as log_2_ fold changes relative to healthy no sn controls. Values represent mean ± SD (*n* ≥ 3).

In Arlo cells, Western blot analysis showed only minor E-cadherin degradation following exposure to wt PAO1 sn, with no significant differences between conditions (Figure 2D,E). While direct LasB effects appear limited, in the presence of live bacteria, additional virulence mechanisms such as Type III Secretion System (T3SS) could facilitate epithelial disruption and enhance LasB accessibility to junctional targets. CLSM imaging showed weak E-cadherin signals in all conditions, with punctate structures in wt PAO1 sn-treated cells (Figure 2F) possibly representing cleaved fragments or indicate E-cadherin reorganization in response to cellular stress. Less pronounced E-cadherin cleavage in Arlo cells compared to Calu-3 may reflect differences in E-cadherin expression, stability, or post-translational modifications, as well as Arlo cells’ tighter junctions and higher TEER, which could limit LasB access to junctional proteins.^17^ Arlo cells may also activate stress responses that protect or reorganize E-cadherin, contributing to their relative resilience.

These findings highlight the differential responses of bronchial (Calu-3) and alveolar (Arlo) epithelial cells to LasB-induced stress, emphasizing the importance of post-translational mechanisms in maintaining epithelial barrier integrity. Cell type-specific responses underscore the complexity of host–pathogen interactions in *P. aeruginosa* infections and suggest that targeted therapeutic strategies may need to account for these tissue-specific differences to maximize effectiveness.

#### 2.1.4 LasB Modulates Cytokine Expression and Cleavage to Suppress Immunity and Induce Tissue Damage

LasB degrades inflammatory cytokines that are essential for immune coordination during infection.^8,31,32^ Cytometric bead array (CBA) data revealed that challenging both lung cell types with wt PAO1 sn resulted in markedly reduced levels of IL-6 and TNF. This suggests that these cytokines may be degraded by the elastase (Figure 2G). LasB degradation of IL-6 is well characterized; the protease directly cleaves the cytokine, thereby suppressing immune signaling and epithelial repair pathways, as validated by protease-specific assays.^32,33^ While an earlier study suggested that *P. aeruginosa* elastase cleaves TNF, the specific contribution of LasB is unclear.^31^ In our assay, TNF levels were significantly reduced in the presence of LasB, supporting the hypothesis that LasB contributes to its degradation during infection. Notably, cells challenged with Δ*lasB* PAO1 sn exhibited higher levels of most cytokines, except IL-1β, which showed slightly reduced activation compared to wt PAO1 sn-treated cells (Figure 2G). This suggests that LasB selectively modulates cytokine activity by suppressing certain mediators while promoting IL-1β maturation.^34,35^ Furthermore, these findings indicate that LasB-induced barrier disruption is primarily attributed to its proteolytic activity on junctional proteins rather than inflammatory processes, as it occurs independently of pro-inflammatory cytokine signaling.

Gene expression analyses revealed distinct patterns between Calu-3 and Arlo cells. Overall pro-inflammatory cytokine expression was significantly higher in Calu-3 cells. In these cells, wt PAO1 sn induced a stronger upregulation of *TNF*, *IL1A*, *IL1B*, and *CXCL8* compared to Δ*lasB* PAO1 sn, even though *TNF* and *IL6* were diminished at the protein level (Figure 2H). This discrepancy suggests transcriptional responses may compensate for LasB-associated reductions in cytokine protein abundance. The robust inflammatory response in Calu-3 cells aligns with their anatomical role as bronchial epithelial cells, which are evolutionarily primed to initiate immune signaling through cytokine/chemokine production and interaction with submucosal immune cells like macrophages and dendritic cells.^36,37^ This is supported by studies showing that Calu-3 cells actively secrete IL-6, CXCL8, and IL-10 during infection, initiating inflammatory cascades.^38,39^

In contrast, Arlo cells showed a different cytokine expression pattern (Figure 2H). *TNF* expression remained comparable between wt PAO1 sn and Δ*lasB* PAO1 sn, while *IL1A* was more upregulated in wt PAO1 sn-treated cells. Conversely, *CXCL8* and *IL1B* were more strongly upregulated in Δ*lasB* PAO1 sn-treated cells. These differences likely reflect cell line-specific properties, as different model systems can exhibit distinct immune-related responses, making it challenging to define clinically relevant pathways. Additionally, the more restrained inflammatory signaling observed in Arlo cells may be due in part to the absence of immune cells, such as alveolar macrophages, which are known to amplify cytokine responses in vivo.^40^ Our findings are consistent with studies highlighting the crucial role of LasB in immune evasion, allowing chronic colonization.^11,32^ By reducing levels of cytokines such as IL-6 and simultaneously activating others like IL-1β, LasB creates a balanced environment that suppresses immunity while inducing localized tissue damage, potentially facilitating bacterial persistence.

To examine how LasB modulates anti-inflammatory signaling, we assessed the expression of *IL4*, *IL13*, *IL37*, and *TGFB1* in both cells. Most of these cytokines showed negligible expression, with *TGFB1* being the only one consistently expressed across all samples. However, exposure of cells to wt PAO1 sn did not alter *TGFB1* or any other tested anti-inflammatory cytokines, suggesting that its primary role is to modulate pro-inflammatory mediators rather than directly induce an anti-inflammatory response.

The impact of LasB extends beyond inflammatory cytokines and also includes colony-stimulating factors (CSFs), which are crucial for myeloid cell function.^41^ Our data reveal that LasB induced a marked reduction in both GM-CSF (encoded by *CSF2*) and G-CSF (encoded by *CSF3*) levels in bronchial Calu-3 cells, suggesting that LasB may contribute to the degradation of these cytokines (Figure 2G). Notably, previous in vivo studies have reported reduced G-CSF levels as a consequence of LasB activity in mice, supporting our findings and highlighting the relevance of this mechanism in disease models.^11^ Interestingly, Calu-3 cells showed differential regulation of these CSFs when challenged with wt PAO1 sn versus Δ*lasB* PAO1 sn. *CSF2* upregulation was lower upon infection with Δ*lasB* PAO1 sn, while *CSF3* showed higher upregulation, suggesting that LasB promotes GM-CSF production but inhibits G-CSF protein levels (Figure 2H). This complex modulation of CSFs by LasB aligns with its established role in immune evasion and early infection stages, particularly its higher activity in initial colonization. Additionally, LasB is able to diminish not only junctional proteins, but also various host immune factors.^32,42^ These results indicate a sophisticated mechanism by which *P. aeruginosa* manipulates the response of host myeloid cells and possibly influences the function of neutrophils and macrophages during the early phase of infection.^43,44^ Our findings underline the multifaceted role of LasB in the pathogenesis of *P. aeruginosa* and its potential as a therapeutic target to prevent initial colonization.^45^

The contrasting protein and gene expression profiles observed in our study underscore the complexity of host–pathogen interactions, where transcriptional responses may act to counterbalance protease activity. This dual mechanism provides insight into why Δ*lasB* strains, despite inducing stronger immune activation, exhibit reduced chronic colonization in vivo.^11,45^ Overall, these findings emphasize the importance of LasB as a therapeutic target in *P. aeruginosa* during early stages of infection, prompting further exploration of antivirulence strategies targeting this multifaceted virulence factor.

### 2.2 Inhibition of LasB-Induced Virulence: Restoring Epithelial Integrity

To evaluate the potential of antivirulence strategies in mitigating LasB-induced barrier dysfunction, we tested three recently published phosphonate-based LasB inhibitors (compounds **1**–**3**) (Table S1).^23,46^ These compounds have shown high efficacy in vivo and a favorable ADMET profile, highlighting their translational potential.

In Calu-3 cells, all three compounds significantly attenuated LasB-induced TEER reduction at 100 μM. Compounds **1**, **2**, and **3** preserved TEER at approximately 79.7%, 88.6%, and 95.4% of no sn control levels, respectively, compared to the drastic reduction of 14.1% observed in untreated wt PAO1 sn-challenged cells (Figure 3A). This protective effect was observed at 10 and 1 μM, with slight dose-dependency (Figure S5). Paracellular permeability assays using FluNa revealed that compound-treated cells exposed to wt PAO1 sn exhibited permeability comparable to cells treated with Δ*lasB* PAO1 sn, indicating effective barrier preservation (Figure 3B).

**Figure 3:**
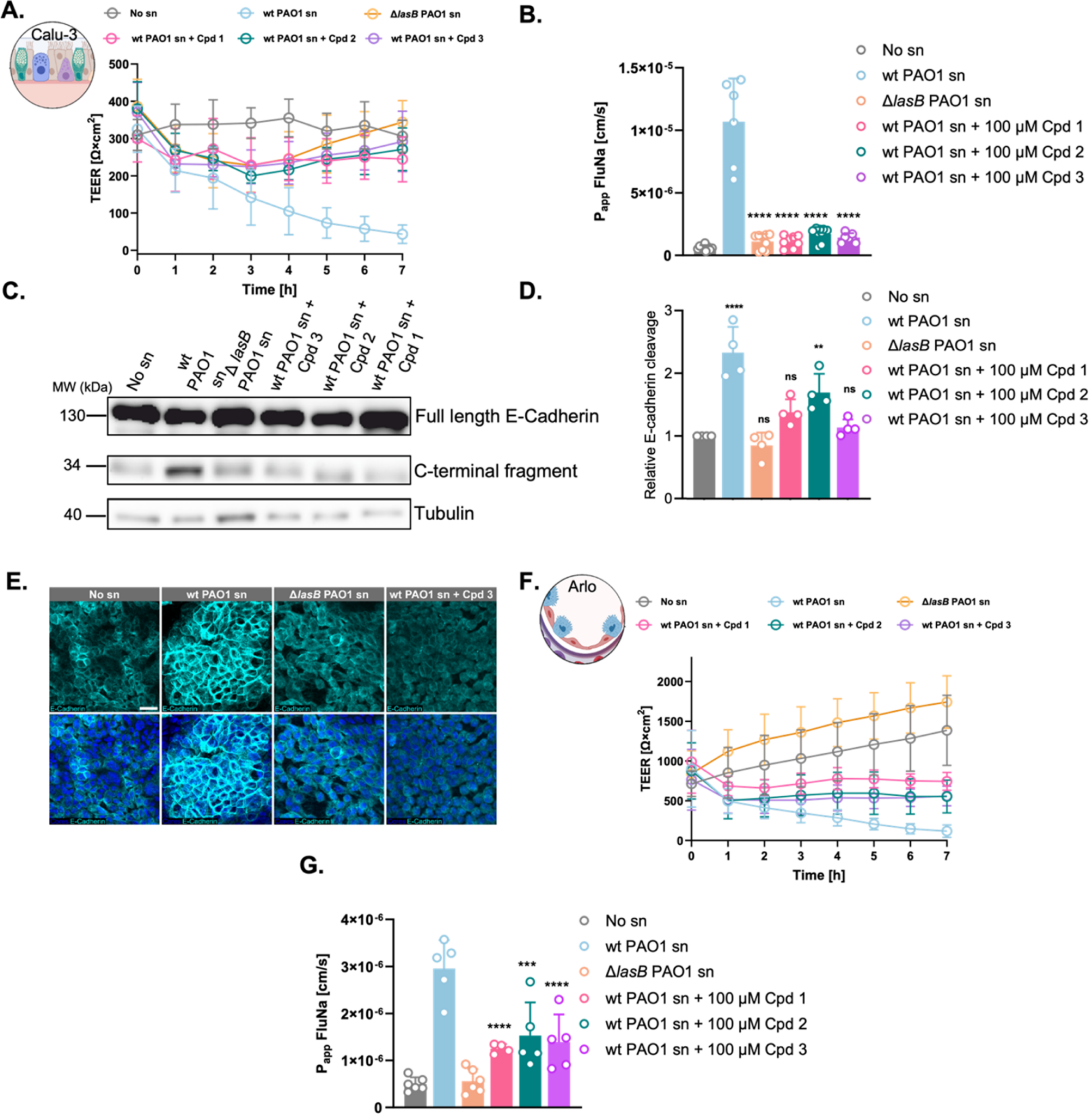
Pharmacological inhibition of LasB protects epithelial barrier function and prevents E-cadherin degradation. (A) Transepithelial electrical resistance (TEER) and (B) paracellular permeability (P_app_) to fluorescent sodium (FluNa) in Calu-3 cells under healthy conditions or following challenge with wild-type (wt) *Pseudomonas aeruginosa* PAO1 supernatant (sn), Δ*lasB* PAO1 sn, or wt PAO1 sn with 100 μM of LasB inhibitors (**1**–**3**). (C) Western blot analysis of E-cadherin cleavage in Calu-3 cells across the same treatment groups, showing full-length and C-terminal fragment bands alongside tubulin as a loading control. (D) Relative quantification of E-cadherin cleavage in Calu-3 cells. Data represent mean ± SD (*n* = 3). Statistical analysis was performed using one-way ANOVA with Dunnett’s multiple comparisons test (****p < 0.0001; **p < 0.01; ns, not significant). (E) CLSM visualization of E-cadherin (cyan) localization in Calu-3 cells under no sn control, wt PAO1 sn, Δ*lasB* PAO1 sn, and wt PAO1 sn + compound **3** conditions. Nuclei were counterstained with DAPI (blue). Scale bar, 25 μm. (F) TEER and (G) FluNa permeability in Arlo cells under identical conditions as Calu-3 cells.

In Arlo cells with tighter junctions, the protective effect was more modest. TEER values were maintained at 53.6% with compound **1** and 40.4% with compounds **2** and **3** when challenged with wt PAO1 sn, compared to the severe reduction to 8.4% seen in untreated cells (Figure 3F). While these compounds effectively enhanced epithelial barrier function in both cell types, their impact was more pronounced in Calu-3 cells. This difference likely reflects Arlo cells’ stronger baseline barrier integrity, which may limit further improvements in TEER upon treatment. Nonetheless, significant reductions in paracellular permeability were observed in both cell types following compound treatment, indicating effective barrier restoration (Figure 3B,G). Notably, Arlo cells challenged with Δ*lasB* PAO1 sn showed progressive increases in TEER, suggesting engagement of compensatory mechanisms to restore barrier function in the absence of LasB (Figure 3F). Similarly, healthy Arlo cells also exhibited gradual TEER increases during prolonged culture, likely reflecting tight junction maturation amplified under Δ*lasB* PAO1 sn-treated conditions. Together, these findings underscore the crucial role of LasB in disrupting epithelial integrity. To ensure specificity, we tested the compounds against Δ*lasB* PAO1 sn and assessed their potential cytotoxicity (Figure S6). Neither TEER nor permeability assays revealed any off-target effects or cytotoxicity, confirming that barrier preservation was due to specific LasB inhibition. LDH release measurements further validated the noninvasive nature of the TEER assay and the absence of cytotoxicity (Figure S7).

Western blot analysis confirmed our previous observations, showing pronounced E-cadherin C-terminal fragments in wt PAO1 sn treated Calu-3 cells compared to no sn and Δ*lasB* PAO1 sn-treated controls (Figure 3C). Treatment with each compound at 100 μM effectively inhibited LasB-induced E-cadherin degradation with the resulting C-terminal fragment levels comparable to the controls. Quantification of E-cadherin cleavage further corroborated these findings, with compound **3** showing the highest efficacy, demonstrating that the compounds mitigate LasB-induced E-cadherin degradation and preserve epithelial barrier integrity (Figure 3D). Arlo cells did not show significant differences in E-cadherin cleavage upon compound treatment, likely due to their inherently stronger barrier properties that make them less susceptible to LasB-mediated barrier disruption (Figure S8). Consequently, inhibitor effects are less pronounced and harder to detect compared to Calu-3 cells.

CLSM analysis of Calu-3 cells provided further insights into the protective effects of our inhibitors against LasB-induced E-cadherin degradation. CLSM imaging revealed that all compounds at 100 μM restored E-cadherin signal patterns to resemble healthy controls, in contrast to the altered distribution observed in wt PAO1 sn-treated cells (Figure 3E–S9A). Similarly, CLSM analysis of Arlo cells mirrored the findings from Western blotting, with no notable changes in E-cadherin distribution compared to controls, consistent with the limited susceptibility of Arlo cells to LasB-mediated cleavage (Figure S9B). Control experiments confirmed that the compounds alone did not affect E-cadherin distribution in either cell type (Figure S9C,D). Moreover, combination treatments with Δ*lasB* PAO1 sn and compounds showed no significant changes in E-cadherin localization, further supporting the specificity of the compounds in targeting LasB-mediated E-cadherin degradation (Figure S9E,F).

Gene expression analysis revealed distinct, cell type-specific cytokine responses to LasB inhibitors. In Calu-3 cells, treatment with the inhibitors partially reversed LasB-induced transcriptional upregulation of several cytokines, most notably *IL1B*, *TNF*, and *IL1A*, with compound **1** showing the strongest effects (Figure 4A, left). Conversely, Arlo cells exhibited further upregulation of most inflammatory cytokines upon compound treatment in wt PAO1 sn challenged cells (Figure 4A, right). This paradoxical response may reflect cell type-specific immune reprogramming, where inhibition of LasB exposes alternative pro-inflammatory signaling cascades.

**Figure 4:**
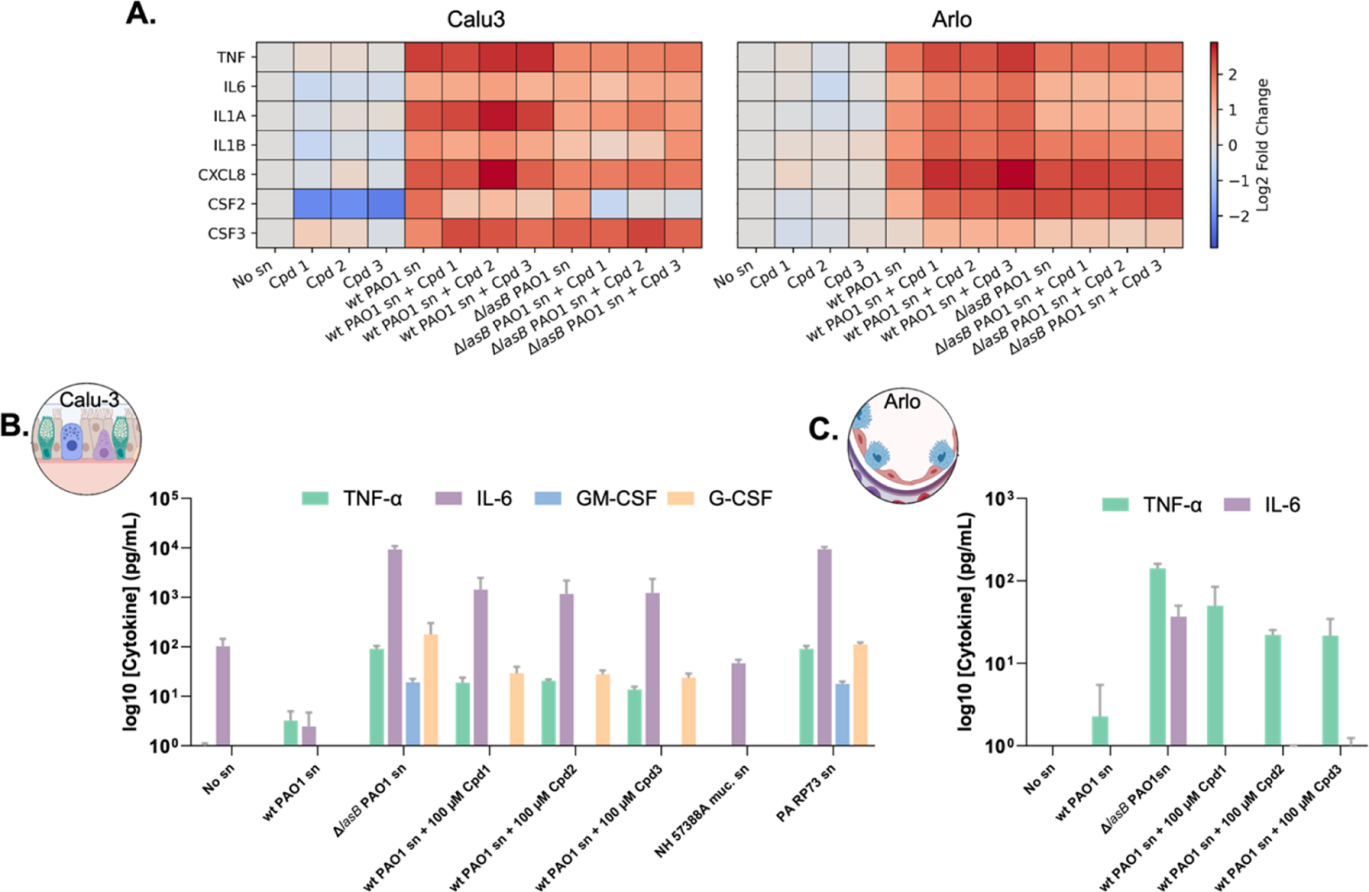
LasB inhibitors differentially modulate cytokine expression and secretion in a cell type-specific manner. (A) Heatmaps depicting log_2_ fold change in mRNA expression of inflammatory cytokines and colony stimulating factors (CSFs) in Calu-3 (left) and Arlo (right) cells under indicated treatment conditions. Cells were either healthy (no supernatant (sn)), treated with compounds alone (compounds **1**–**3**), challenged with wild-type (wt) *Pseudomonas aeruginosa* PAO1 sn, or cotreated with wt PAO1 sn and individual compounds. Additional conditions included Δ*lasB* PAO1 sn alone and in combination with compounds. (B, C) Log_10_ concentrations (pg/mL) of secreted cytokines quantified via cytometric bead array (CBA) in Calu-3 (B) and Arlo (C) cells under the same treatment conditions. Additional conditions in Calu-3 cells include exposure to NH57388A muc. and RP73 sn, representing low- and negligible-LasB-producing clinical isolates, respectively. Cytokine quantification was limited to detectable proteins: TNF, IL-6, GM-CSF, and G-CSF in Calu-3 cells; TNF and IL-6 in Arlo cells. Data represent mean values from ≥3 independent experiments.

CBA analysis demonstrated significantly reduced levels of IL-6 and TNF by wt PAO1 sn, with partial rescue by LasB inhibitors (Figure 4B, C). Furthermore, in Calu-3 cells, evaluation of CSFs showed compound-mediated downregulation of *CSF2* and partial *CSF3* reduction at the transcriptional level (Figure 4A, left). While CBA revealed partial G-CSF protein rescue, GM-CSF remained undetectable, suggesting that transcriptional suppression may dominate over post-translational degradation for this CSF (Figure 4B). Importantly, treatment with compounds alone or in combination with Δ*lasB* PAO1 sn did not alter cytokine secretion in either cell type, confirming specificity of the observed effects (Figure S10).

Further evaluation of cytokine levels in Calu-3 cells challenged with low-LasB-producing strains (NH57388A muc. and RP73) demonstrated that even minimal LasB quantities reduce cytokine levels, consistent with LasB’s proposed role in modulating cytokine stability (Figure 4B). Marked cytokine reduction was observed in cells exposed to NH57388A muc. sn (∼6 nM LasB), producing low LasB levels. In contrast, cytokine levels in cells treated with RP73 sn, a strain with negligible *lasB* expression (LasB <3 nM), were similar to those seen upon exposure to Δ*lasB* PAO1 sn (Figure 4B). These results support the notion that low-level LasB activity may be sufficient to alter the inflammatory milieu during infection. Supporting this notion, experiments using pure LasB on Calu-3 cells revealed that concentrations ranging from 100 to 3 nM effectively diminished cytokines levels. Notably, G-CSF, GM-CSF, or TNF became undetectable, while low levels of IL-6 persisted following 3 nM LasB treatment. This suggests that LasB efficiently targets multiple cytokines, with IL-6 showing relative resistance at lower concentrations. The ability of LasB to reduce cytokine levels at such low concentrations underscores its potential to influence immune signaling during *P. aeruginosa* infections.^32,42^ These findings align with prior reports implicating LasB in host defense disruption, but also suggest the potential limitation of partial inhibition strategies, as residual LasB may still affect key immune mediators.

These results highlight two key challenges: LasB’s extreme proteolytic efficiency necessitates near-complete inhibition to preserve cytokines, and cell type-specific responses complicate therapeutic predictions. To address these complexities and identify broader mechanisms of LasB-mediated immune evasion, we interrogated global transcriptional changes induced by infection and upon treatment.

### 2.3 Transcriptomic Profiling of LasB Effects on Calu-3 Cells

To identify LasB-affected targets and separate them from global *P. aeruginosa*-induced transcriptomic changes in Calu-3 cells, we conducted RNA sequencing. Cells were exposed to conditions differing in the presence, absence, and inhibition of LasB: wt PAO1 sn, Δ*lasB* PAO1 sn, wt PAO1 sn with compound treatment, including compound toxicity controls, and off-target controls (compound + Δ*lasB* PAO1 sn). The resulting data were processed using RNADetector, and differential expression (edgeR) analysis comparing all conditions to wt PAO1 sn-treated samples.^47^ This approach allowed us to identify expression patterns dysregulated by LasB and evaluate the inhibitor’s ability to restore normal expression patterns (Figure 5A,B).

**Figure 5:**
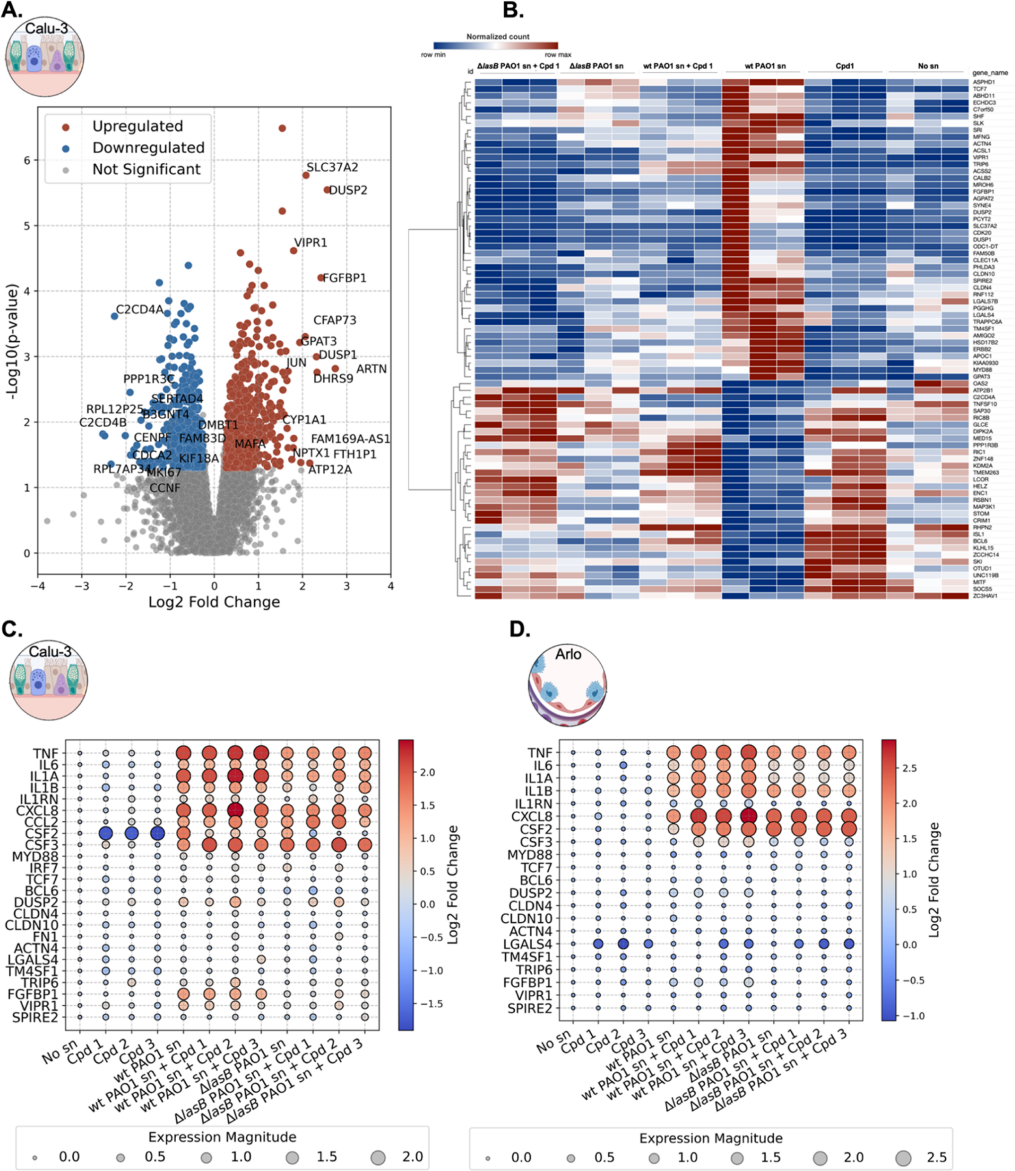
Transcriptomic analysis of LasB-specific effects on lung epithelial cells. (A) Volcano plot of RNA-sequencing data comparing all treatment and control conditions against the wild-type (wt) *Pseudomonas aeruginosa* PAO1 supernatant (sn)-treated Calu-3 cells. Data of the log_2_ fold change is plotted against the -log_10_ p-value. Upregulated genes are shown in red, downregulated genes in blue, and nonsignificant genes in gray. (B) Heatmap showing top upregulated and downregulated genes in wt PAO1 sn-treated cells, with gene expression patterns across all tested conditions, including healthy (no sn), compound **1**, and Δ*lasB* PAO1 sn ± compound **1**. Heatmap generated with Morpheus. (C,D) Bubble heatmaps of qPCR validation in Calu-3 (C) and Arlo (D) cells for selected genes, including cytokines and top LasB-regulated genes. Bubble size represents magnitude of fold change, color indicates direction (red = upregulated, blue = downregulated). Gene expression is normalized to healthy controls. Data are from at least three independent experiments.

Among the significantly affected pathways in Calu-3 cells following LasB treatment, we identified processes previously associated with *P. aeruginosa* pathomechanisms. Upregulation of lipid metabolism genes Acyl-CoA synthetase long chain family member 1 and 1-Acylglycerol-3-phosphate *O*-acyltransferase 2 (*ACSL1*, *AGPAT2*) as well as cell adhesion molecules Claudin-4 and Claudin-10 (*CLDN4*/*10*) suggests cell membrane and barrier alterations, consistent with *P. aeruginosa*’s known disruption of epithelial barriers.^48–50^ Immune modulation was evident through downregulation of 2′-5′-Oligoadenylate Synthetase 2 (*OAS2*) and Tumor Necrosis Factor Superfamily Member 10 (*TNFSF10*) alongside upregulation of Myeloid Differentiation Primary Response 88 (*MYD88*), a key adaptor in innate immune signaling.^51–53^ Further analysis revealed that the dysregulation extends to the upregulation of dual-specificity phosphatases (*DUSP*s), which are involved in Mitogen-Activated Protein Kinase (MAPK) signaling regulation, and downregulation of B-Cell Lymphoma 6 (*BCL6*), a transcriptional repressor in immune responses.^54,55^ Altered expression of transcription factors and signaling molecules Transcription Factor 7 (*TCF7*), Erb-B2 Receptor Tyrosine Kinase 2 (*ERBB2*), and Mitogen-Activated Protein Kinase Kinase Kinase 1 (*MAP3K1*) indicates broad changes in cellular signaling pathways.^56–58^ Notably, upregulation of Vasoactive Intestinal Peptide Receptor (*VIPR1*) and Spire-Type Actin Nucleation Factor 2 (*SPIRE2*), involved in actin organization, suggests LasB-induced changes in cellular signaling and cytoskeletal remodeling.^59,60^ The modulation of growth factor-related genes such as Fibroblast Growth Factor Binding Protein 1 (*FGFBP1*), and cytoskeletal components including Actinin Alpha 4 (*ACTN4*) points to LasB’s interference with normal growth, differentiation, and structural integrity of the airway epithelium.^61,62^ Additionally, the upregulation of Transmembrane 4 L Six Family Member 1 (*TM4SF1*), involved in cell motility, and Thyroid Hormone Receptor Interactor 6 (*TRIP6*), a focal adhesion protein, highlights LasB effects on cell–cell and cell–matrix interactions.^63,64^

Collectively, these data reveal the multifaceted impact of LasB on airway epithelial cells, disrupting barrier integrity, immune signaling, and cellular architecture — hallmarks of *P. aeruginosa* pathogenesis (Figure 5A,B). However, they also highlight the challenge of identifying specific clinical markers to distinguish LasB-specific effects in complex *P. aeruginosa* infections, especially in vivo with multiple affected cell types.

Building on the transcriptomic analysis, we further validated our RNA-seq findings in a Calu-3 based in vitro system through qPCR quantification of selected transcripts: *VIPR1*, *DUSP2*, *SPIRE2*, *CLDN4*, *CLDN10*, *FGFBP1*, *LGALS4*, *MYD88*, *TCF7*, *ACTN4*, *TM4SF1*, *TRIP6*, and *BCL6*. This selection encompassed genes involved in diverse cellular processes, including signaling, cytoskeletal organization, cell adhesion, and immune response, providing a comprehensive view of the impact of LasB (Figure 5C). Among the validated transcripts, *FGFBP1*, *VIPR1*, and *DUSP2* showed the most pronounced upregulation in response to LasB. Notably, *FGFBP1*, a secreted chaperone enhancing FGF signaling and is implicated in angiogenesis, inflammation, and wound healing, was markedly upregulated, suggesting a possible role in tissue repair and remodeling in response to *P. aeruginosa*-induced epithelial damage. Validation in Arlo cells showed overall smaller gene expression changes, with modest upregulation of *DUSP2* and *FGFBP1*, indicating some LasB responsiveness in this cell type (Figure 5D).

Importantly, LasB inhibitors effectively reversed the upregulation of *FGFBP1*, *VIPR1*, and *DUSP2* in Calu-3 cells, with compound **3** showing the strongest efficacy (Figure 5C). This confirms the inhibitors’ specificity, demonstrates LasB-dependent changes in gene expression, and highlights the therapeutic potential of targeted LasB inhibition in preserving epithelial function.

#### 2.3.1 Comparative Analysis and Insights into LasB-Mediated Barrier Disruption

Since in vitro screening can be strongly influenced by cell type, we compared our results in silico with all public transcriptomic data sets from similar *P. aeruginosa* infection models to place our findings in a broader biological context. FastQ files from experiments with comparable time points and experimental conditions (controls and infected cells) were retrieved using Sequence Read Archive (SRA) tools from the Gene Expression Omnibus (GEO) database — specifically, GSE97036 (6 h, no arsenite) and the normal lung epithelial cell line (NuLi-1) from GSE199424 (8 h) (see Data S1 for used SRA files).^65^ These data sets were processed together with our RNA sequencing data using a common pipeline to ensure normalization across all samples (Figure S11). While differences in sequencing depth and library preparation can introduce variability, differential expression analysis revealed consistent expression patterns across models. Notably, *DUSP2* was repeatedly upregulated in both Calu-3 and primary bronchial epithelial cells upon infection, suggesting that *DUSP2*, a key regulator of MAPK signaling, plays a conserved role in epithelial responses to *P. aeruginosa*, likely contributing to inflammation or stress pathway modulation, and is unlikely to represent a cell line–specific artifact.

Claudin-4 was identified as a potential LasB target in Calu-3 cells. CLSM imaging revealed accumulation or redistribution of Claudin-4 signal in Calu-3 cells treated with LasB, suggesting altered junctional integrity or protein localization (Figure 6A). This effect was less apparent in Arlo cells, suggesting cell type-specific differences or the need for higher LasB concentrations or prolonged incubation (Figure 6B). Moreover, Claudin-4 has previously been implicated as a LasB target in nasal epithelial cells, further supporting its relevance as a potential proteolytic substrate in our model.^66^ Importantly, junctional disruption was mitigated by LasB inhibitors, demonstrating their efficacy in preserving Calu-3 barrier integrity (Figures 6A and S12A). This finding validates our transcriptomic analysis and uncovers a mechanism by which LasB may compromise bronchial airway epithelial barriers. Furthermore, all inhibitors (compounds **1**–**3**) were applied to Calu-3 and Arlo samples under each treatment condition (no sn, Δ*lasB* PAO1 sn, and wt PAO1 sn) to not only test LasB inhibition but also their potential side effects on Claudin-4 localization (Figure S12). CLSM confirmed that every compound inhibits LasB, resulting in the preservation of Claudin-4 localization in Calu-3 cells (Figure S12A). Compounds alone did not alter tight junction protein expression (Figure S12C,D) and showed no major impact in combination with Δ*lasB* PAO1 sn (Figure S12E,F). This underscores the inhibitors’ specificity in protecting proteins critical for cell–cell interaction.

**Figure 6:**
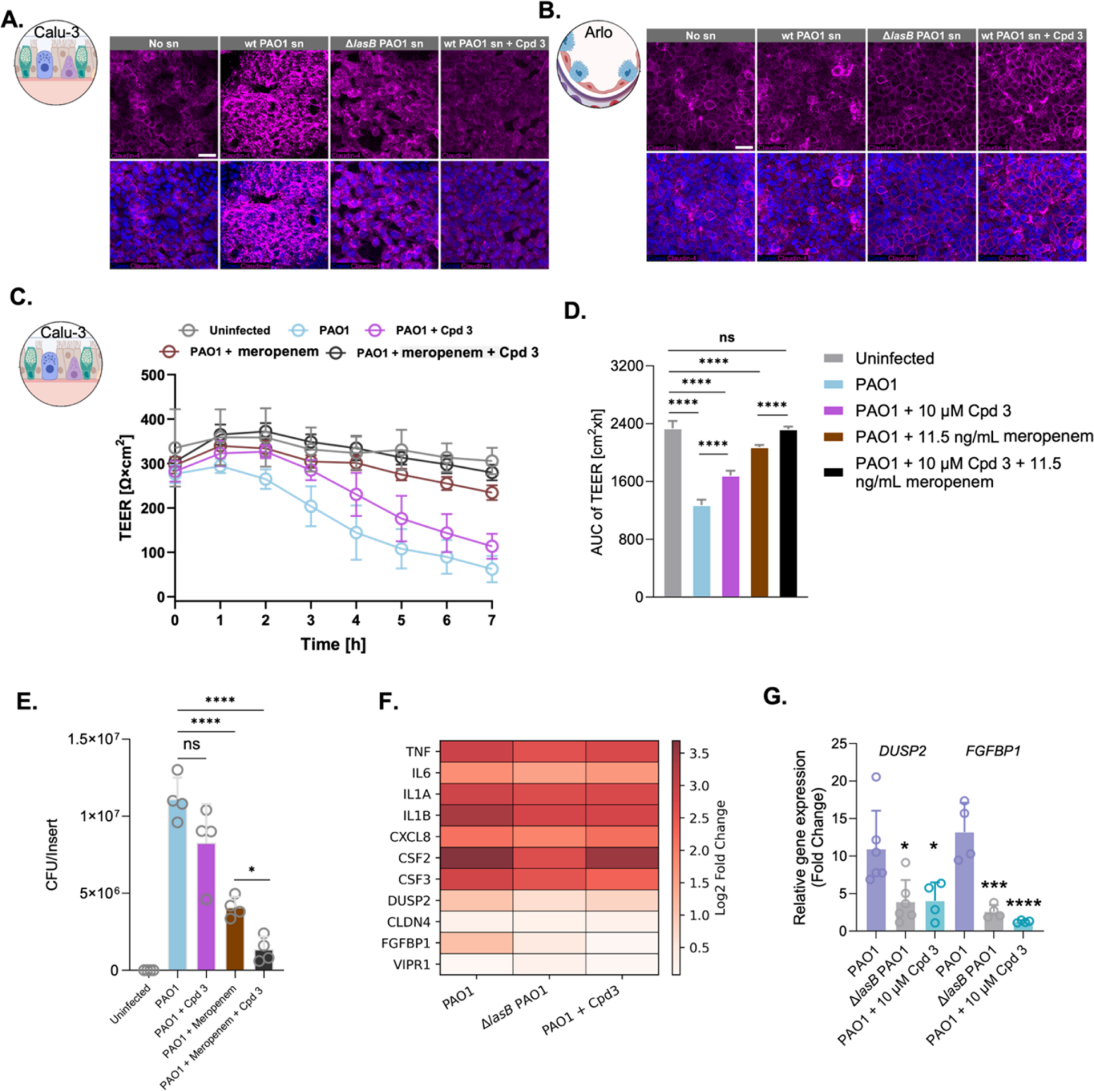
LasB inhibition preserves epithelial integrity and modulates host responses during live *Pseudomonas aeruginosa* infection. (A,B) Confocal laser scanning microscopy (CLSM) images showing Claudin-4 (magenta) localization in Calu-3 (A) and Arlo (B) cells under different treatment conditions (healthy control, wild type (wt) PAO1 supernatant (sn), Δ*lasB* PAO1 sn, and wt PAO1 sn + compound **3**). Nuclei were counterstained with DAPI (blue). Scale bar: 25 μm. (C) Transepithelial electrical resistance (TEER) measurements of Calu-3 monolayers infected with wt or Δ*lasB* PAO1 at multiplicity of infection (MOI) of 30, treated with Meropenem (11.5 ng/mL), compound **3** (10 μM), or their combination. (D) Area under the curve (AUC) quantification of TEER data from panel C further emphasizing the protective effect of combined treatment. (E) Colony-forming unit (CFU) counts from infected Calu-3 cells under corresponding conditions. (F) Heatmap showing relative expression of inflammatory cytokines and colony-stimulating factors alongside transcriptomic markers in Calu-3 cells following infection and treatment. (G) Relative gene expression (fold change) of *DUSP2* and *FGFBP1* under the same conditions. Data represent means from *n* = 3 independent experiments. Statistical analysis was performed using one-way ANOVA followed by Dunnett’s multiple comparisons test (****p < 0.0001; ***p < 0.001; **p < 0.01; ns, not significant).

#### 2.3.2 Toward a Physiologically More Relevant Infection Model

To further elucidate the role of LasB in a physiologically more relevant context, we extended our model from bacterial supernatants to live *P. aeruginosa* infections, capturing real-time host–pathogen interactions and LasB production. This approach allowed evaluation of our LasB inhibitors in a more complex setting, uncovering additional aspects of host–pathogen interactions and the role of LasB in virulence.

In this context, Calu-3 cells were infected with wt or Δ*lasB* PAO1. A clear dose-dependent toxicity of wt PAO1 was observed after challenging the lung cells with a multiplicity of infection (MOI) of 3 to 300, whereas Δ*lasB* PAO1 showed no toxicity and slightly increased TEER at the same MOI range, suggesting protective epithelial responses, possibly via enhanced tight junctions or innate immune activation in the absence of major virulence factors (Figure S13). Area-under-the-curve (AUC) analysis emphasized the dose-dependent nature of wt PAO1-induced damage (Figure S13), and LDH release at MOIs of 30 and 300 confirmed negligible cell death (Figure S14).

We evaluated compound **3** at 10 μM in mitigating barrier disruption during *P. aeruginosa* PAO1 infection at MOI 30 compared to Meropenem treatment at 11.5 ng/mL, a concentration approximately 200-fold lower than the minimum inhibitory concentration (MIC) previously determined under similar experimental conditions (2 ± 1 μg/mL in 0.5% LB and 99.5% HBSS). This sub-MIC concentration was deliberately selected to prevent complete bacterial killing, thereby enabling assessment of compound **3**’s protective effects on epithelial barrier integrity in the presence of viable bacteria. In addition, we evaluated the combination of both compounds for potential synergistic effects (Figure 6C,D).

Wt PAO1 infection reduced TEER to ∼20% of healthy control levels after 7 h. Compound **3** partially preserved TEER at ∼37% of the uninfected control, while sub-MIC Meropenem was more effective in maintaining epithelial integrity, with TEER levels reaching ∼77% of the uninfected control. Remarkably, the combination of compound **3** and Meropenem improved TEER preservation to ∼92% of healthy control levels, exceeding the effect of each treatment individually. This effect likely arises from the complementary mechanisms of action: while Meropenem reduces the bacterial load, compound **3** attenuates the virulence activity of LasB, thereby preserving barrier integrity and potentially improving antibiotic penetration. These findings highlight the therapeutic potential of antivirulence strategies in conjunction with conventional antibiotics, even at sub-MIC levels (Figure 6C,D).

Colony-forming unit (CFU) counts provided further insights into bacterial survival and growth (Figure 6E). Treatment with compound **3** alone slightly reduced CFUs compared to untreated wt PAO1-infected cells, consistent with its role as an antivirulence agent rather than a bactericidal compound. In contrast, Meropenem reduced bacterial burden significantly, especially in combination with compound **3**. Our data support pairing LasB inhibitors with antibiotics to enhance clearance and preserve barrier function, particularly in antibiotic-resistant contexts. This strategy may be especially beneficial in immunocompromised patients, where antivirulence treatments alone may be insufficient.

#### 2.3.3 Host Response Modulation and Transcriptomic Validation by LasB Inhibition

To assess host response modulation during live *P. aeruginosa* infection, we evaluated a broad panel of inflammatory and transcriptomic markers in Calu-3 cells. These included pro-inflammatory cytokines, CSFs, and key transcriptomic targets identified in earlier analyses: *DUSP2*, *CLDN4*, *FGFBP1*, and *VIPR1* (Figure 6F), thereby providing a comprehensive overview of host transcriptional responses to infection.

Wt PAO1 infection induced strong upregulation of all measured cytokines and CSFs, reflecting a strong pro-inflammatory response. Δ*lasB* PAO1 infections showed attenuated elevation, confirming LasB as a key driver of excessive inflammatory signaling. Treatment with compound **3** during wt PAO1 infection substantially reversed this effect. For *IL6*, *IL1B*, and *CSF3*, expression levels approached levels comparable to Δ*lasB* PAO1-infected condition, indicating mitigation of LasB-driven inflammation during bacterial infections.

Within this broader context, *DUSP2* and *FGFBP1* were particularly responsive to LasB. These genes were strongly upregulated during wt PAO1 infection, but their expression was significantly lower in Δ*lasB*-infected cells and upon compound **3** treatment, consistent with RNA-seq data (Figure 6E,G). The consistent regulation of *FGFBP1* may point to its involvement in epithelial repair or remodeling, while *DUSP2* likely reflects an activation of stress or inflammatory pathways. The ability of compound **3** to normalize the expression of both genes highlights its ability to restore host-cell homeostasis and counteract LasB-induced dysregulation.

Together, these findings establish LasB not only as a critical virulence factor driving inflammation and transcriptional reprogramming in host cells but also as a viable therapeutic target. Broad transcriptional normalization observed with compound **3** reinforces antivirulence strategies as effective adjuncts to preserve epithelial integrity during *P. aeruginosa* infection.

## 3 Conclusions

This study provides a comprehensive analysis of the multifaceted role of LasB in *P. aeruginosa* pathogenesis, revealing its significant impact on epithelial barrier integrity, immune modulation, and gene expression. Our findings demonstrate LasB-mediated cleavage of E-cadherin, disruption of Claudin-4 integrity, modulation of inflammatory responses, and widespread gene expression changes, particularly in *FGFBP1* and *DUSP2*. While Claudin-4 has been studied in other epithelial contexts, including nasal epithelial cells, our work newly establish its altered localization in bronchial airway epithelia in response to LasB activity, extending its relevance as a lower respiratory tract virulence target.^66^ Furthermore, we demonstrate that LasB reduces colony-stimulating factors GM-CSF and G-CSF levels, providing novel insights into its immune-modulatory functions consistent with prior in vivo studies in murine models.^11^ Importantly, our study provides direct evidence that LasB diminishes GM-CSF and G-CSF levels in human bronchial epithelial cells.

The efficacy of our LasB inhibitors in mitigating these effects, especially when combined with antibiotics, highlights the potential of antivirulence strategies in combating *P. aeruginosa* infections. The enhanced effect of LasB inhibitors as an adjunct to antibiotics in live bacterial infection model is particularly promising, suggesting an approach to improve treatment efficacy while limiting antimicrobial resistance.^23^ Recent in vivo studies supports this notion, showing LasB inhibitors reduce bacterial burden and improve antibiotic performance in animal models.^9,23,46^

Our transcriptomic analysis has uncovered novel targets and pathways affected by LasB, offering deeper understanding of *P. aeruginosa* pathogenesis and potential biomarkers for tracking disease progression and treatment response. Given the high attrition in clinical development (>65% fail in phase II), rigorous preclinical studies remain essential to identify promising candidates early in development and derisk potential safety or efficacy issues.^67^ Our integrated approach, combining functional assays, protein analysis, and transcriptomics, provides a strong foundation for future translational research.

Building on these findings, future studies should extend molecular characterization of LasB-mediated effects across the broader secretome to clarify its relative contribution among coregulated virulence factors. Validating key observations under air–liquid interface conditions using advanced airway epithelial models could further strengthen physiological relevance and inform translational applications, including the use of human airway basal epithelial cell line BCi-NS1.1 and donor-derived primary airway epithelia that better recapitulate mucociliary differentiation and native secretory environments. In parallel, long-term evaluation of LasB inhibition, exploration of combination therapies with other antimicrobials (e.g., phages) or antivirulence agents targeting secretion systems or biofilm formation, and optimization of respiratory delivery in chronic infection models, including cystic fibrosis, will be critical for advancing therapeutic translation.^24^

Overall, this work advances understanding of *P. aeruginosa* pathogenesis and demonstrates the potential of targeting virulence factors as a strategy against antibiotic-resistant infections. As antimicrobial resistance grows, innovative antivirulence strategies will be critical for developing effective therapies.

### 3.1 Material and Methods

#### 3.1.1 Cell Cultures

Arlo cells (passages 1–20) were cultured in SAGM (Lonza) supplemented with the SingleQuots pack, 1% fetal calf serum (FCS, Sigma), and 1% penicillin–streptomycin, following the protocol described by Carius et al.^17^ Calu-3 cells were maintained in MEM (Life Technologies) containing 10% FCS, 1% penicillin–streptomycin, 1% nonessential amino acids, and 1% sodium pyruvate. For passaging, cells were washed with PBS, detached using Trypsin/EDTA at 37 °C for 10–20 min, neutralized with fresh medium, centrifuged (300*g*, 5 min), and resuspended in 5 mL medium. Routinely, 2.5 × 10^6^ cells were seeded per T75 flask (13 mL final volume) and cultured at 37 °C, 5% CO_2_, with medium changes every 2–3 days.

#### 3.1.2 Transwell Preparation and Transepithelial Electrical Resistance (TEER) Experiments

For TEER under liquid-covered conditions, Calu-3 and Arlo cells were seeded on 0.33 cm^2^ permeable inserts (400 nm pore size; Corning, 3470) at 3 × 10^4^ and 3.3 × 10^4^ cells per insert, respectively, and cultured for 8–10 days to establish tight junctions. Medium was changed every 2–3 days with fresh MEM or SAGM. TEER was measured using an EVOM2 V-ohmmeter (WPI) with an STX2 electrode after equilibrating cells in HBSS (Ca^2+^/Mg^2+^) for 30–60 min at 37 °C. Test compounds, medium controls (HBSS ± DMSO), blank inserts, and PAO1 supernatant controls were included. Plates were incubated at 37 °C, 5% CO_2_, 200 rpm, and TEER was recorded hourly for up to 7 h, followed by sample collection for further analysis.

#### 3.1.3 Transport Experiments and P_app_ Calculations

FluNa transport across Calu-3 and Arlo monolayers was assessed in parallel with TEER measurements. After equilibration with HBSS and initial TEER recording, 176 μL FluNa (10 μg/mL in HBSS) and 44 μL of test solution were added apically, with 800 μL HBSS basolaterally. Immediately, 20 μL (apical) and 200 μL (basolateral) samples were taken to determine initial concentrations. Plates were incubated at 37 °C, 5% CO_2_ (150 rpm), and 200 μL basolateral samples were collected hourly for 7 h, replaced with fresh HBSS after each sampling. A 1:2 FluNa calibration curve was prepared in duplicate. Fluorescence was measured using a CLARIOstar plate reader (excitation 488 nm, emission 530 nm), and gain was set to the highest calibration signal.

#### 3.1.4 LDH Quantification

LDH release was determined using the CytoTox 96 Assay (Promega) per manufacturer’s instructions. Apical samples (50 μL) were incubated with substrate for 30 min, stopped, and absorbance measured at 490 nm. Blank values were subtracted from all readings.

#### 3.1.5 Gene Expression Analysis in Calu-3 and Arlo Cells

RNA was extracted using the RNeasy Micro Kit (Qiagen) with minor adjustments and eluted in 11 μL RNase-free water. Purity and concentration were measured via NanoDrop 2000. cDNA was synthesized using the High-Capacity cDNA Reverse Transcription Kit (Applied Biosystems), and qPCR was performed with PowerUp SYBR Green Master Mix on a StepOne Plus system. Expression was analyzed by the ΔΔCt method relative to healthy controls.

#### 3.1.6 Cytometric Bead Array (CBA)

Cytokine levels were measured by CBA using BD Human Soluble Protein Flex Sets for IL-6, TNF, G-CSF, MCP-1, and IL-1β with the Master Buffer Kit, following the manufacturer’s protocol. Samples were analyzed on a BD LSRFortessa FACS, and data were processed with FCAP Array v3.0.1 (BD Biosciences).

#### 3.1.7 Quantification of E-Cadherin via Western Blot

The procedure was performed as described by Aljohmani et al. with a modification in the lysis buffer volume.^68^ Cells were lysed in buffer containing Tris·HCl, NaCl, Triton X-100, EDTA, Na_3_VO_4_, PMSF, and 1,10-phenanthroline with Complete Inhibitor (Roche) for 10 min at 4 °C. Lysates were centrifuged (16,000 g, 15 min, 4 °C), and protein concentration was determined using a BCA kit (Thermo Fisher). SDS-PAGE, transfer, immunoblotting, and detection were carried out as described by Aljohmani et al.

#### 3.1.8 Visualization of E-Cadherin and Claudin-4 via Confocal Laser Scanning Microscopy (CLSM)

Arlo and Calu-3 cells were cultured on transwell inserts for 10 days, fixed with 4% paraformaldehyde, permeabilized (1% BSA, 0.05% saponin), and stained with mouse anti-E-Cadherin (1:50) and rabbit anti-Claudin-4 (1:100) antibodies. Alexa Fluor 546 and 488 secondary antibodies and DAPI were applied sequentially. Membranes were mounted with Fluorescence Mounting Medium and imaged using a Leica TC SP8 confocal microscope with a 25× water objective under standard laser and detection settings. Images (2048 × 2048) were acquired sequentially to minimize crosstalk and processed using ImageJ and GIMP.

#### 3.1.9 RNA Sequencing

Calu-3 cells were treated with wt PAO1 sn, Δ*lasB* PAO1 sn, 100 μM compound **1** ± supernatants, and appropriate controls. After 7 h, RNA was extracted (Qiagen Buffer RLT + Monarch Cleanup Kit; RIN >8). Strand-specific mRNA libraries were prepared using the NEBNext Ultra II Directional RNA Library Prep Kit and sequenced on an Illumina NovaSeq 6000 (PE50, ∼30 million reads/sample). Data were processed with RNADetector (using STAR and featureCounts), normalized via edgeR, and analyzed for pathway enrichment with Reactome and PADOG.^69^ Visualizations (volcano plots, heatmaps) were generated in Python (numpy, pandas, matplotlib, seaborn). Selected genes were validated by qPCR.

#### 3.1.10 Calu-3 Transwell-Based Bacterial Infection Model

Wild-type PAO1 and PAO1 Δ*lasB* strains were grown overnight in LB at 37 °C, 180 rpm, and subcultured to OD_600_ ≈ 2. Bacteria were standardized to OD_600_ 1.5 and diluted 1:10 for an MOI of 30. Calu-3 cells were cultured on transwells for 9 days, washed, and equilibrated in HBSS prior to infection. 190 μL HBSS with 10 μL bacterial suspension was added apically and 800 μL HBSS basolaterally. When indicated, compound **3** (0.5% DMSO final) or Meropenem was applied apically. Plates were incubated at 37 °C, 5% CO_2_, and TEER was monitored hourly for 7 h. Apical samples were taken at 0 and 7 h, serially diluted, plated, and CFUs enumerated after incubation.

## Supplementary Material





## Data Availability

All data are available in the main text or the Supporting Information. The data that support the findings of this study are available from the corresponding authors upon reasonable request.

## Notes

Supporting Information. Extended material and methods; supplementary figures (Figures S1–S14); supplementary tables (Tables S1–S3) (PDF)
RNA sequencing metadata and associated analysis files (Data S1) (ZIP)
