# Supplementary material for: Exploring the Role of Pseudomonas aeruginosa Elastase in Lung Epithelial Barrier Dysfunction: Advancing toward Antivirulence Therapies
Source: ACS Infect Dis. 2026 Apr 10;12(5):1611–26. doi: 10.1021/acsinfecdis.5c00915 (PMC13162270; doi:10.1021/acsinfecdis.5c00915)
Supplement: Supplementary file 1 [file id5c00915_si_001.pdf]

Supporting Information

Exploring the Role of *Pseudomonas aeruginosa* Elastase in Lung Epithelial Barrier Dysfunction: Advancing Towards Antivirulence Therapies

Roya Shafiei[+,a,b,d], Alaa Alhayek[*,+,a], Lukas Hiller[a,b,d,e], Lorenz Latta[a], Tobias Neu[a,b,d], Ahmad Aljohmani[c,d], Sahar Abdollahibiroun[a], Eva-Maria Schönborn[a], Daniela Yildiz[c,d], Nicole Schneider-Daum[a], Claus-Michael Lehr[a,b,d], Jörg Haupenthal[a,d,e], Anna K. H. Hirsch[*,a,b,d,e]

[a] R. Shafiei, A. Alhayek, L. Hiller, L, Latta, T. Neu, S. Abdollahibiroun, E-M. Schönborn, N. Schneider-Daum, C-M. Lehr, J. Haupenthal, A. K. H. Hirsch

Helmholtz-Institute for Pharmaceutical Research Saarland (HIPS), Campus E8.1, 66123 Saarbrücken, Germany; Helmholtz Centre for Infection Research (HZI), Inhoffenstraße 7, 38124 Braunschweig, Germany

[b] R. Shafiei, L. Hiller, T. Neu, C-M. Lehr, A. K. H. Hirsch

Department of Pharmacy, Saarland University, 66123 Saarbrücken, Germany

[c] A. Aljohmani, D. Yildiz

Institute of Experimental and Clinical Pharmacology and Toxicology, PZMS, ZHMB, Saarland University, Kirrbergerstraße 100, 66421 Homburg/Saar, Germany

[d] R. Shafiei, L. Hiller, T. Neu, A. Aljohmani, D. Yildiz, C-M. Lehr, J. Haupenthal, A. K. H. Hirsch

PharmaScienceHub (PSH), Campus A2.3, 66123 Saarbrücken, Germany

[e] L. Hiller, J. Haupenthal, A. K. H. Hirsch

German Center for Infection Research (DZIF), Inhoffenstraße 7, 38124 Braunschweig, Germany

[+] Authors contributed equally.

Correspondence to: anna.hirsch@helmholtz-hips.de, alaa.alhayek@unibas.ch

Material and Methods

Preparation of Bacterial Culture Supernatants

Bacterial culture supernatants were obtained from *Pseudomonas aeruginosa* strain PAO1 (DSM-19880) and its *lasB* knockout variant after overnight growth in lysogeny broth (LB) medium. Cultures were incubated at 37°C with shaking at 180 rpm for approximately 18 to 19 h. Following incubation, the cultures were centrifuged at 5000 rpm for 10 min at 4°C. The supernatants were then filtered through 0.2 µm non-pyrogenic sterile filters. Aliquots were prepared for single-use and stored at -80°C until needed.

Cell Cultures

Arlo cells (passage 1–20) were cultured in SAGM™ (Small Airway Epithelial Cell Growth Medium, Lonza) with SingleQuots™ Supplement Pack. The complete medium was additionally supplemented with 1% (v/v) fetal calf serum (FCS, Sigma) and 1% (v/v) penicillin-streptomycin and cells were maintained according to the method described in Carius *et al.*[17] Cells were maintained at 37°C in a humidified 5% CO_2 atmosphere, with medium changes every 2–3 days. Cells were passaged every 7 days: washed twice with PBS (Sigma), detached using 2 mL trypsin-EDTA (0.05%; Gibco) for 8 min, centrifuged at 300 RCF for 4 min, and resuspended in complete SAGM. Flasks were pre-coated for 1 h at 37 °C with 2 mL of 1% (v/v) human fibronectin (1 mg/mL; Corning) and 1% (v/v) bovine collagen type I (3 mg/mL; Sigma) in sterile distilled water. Coating solution was removed immediately before seeding. 0.7 × 10^6 cells were seeded per flask or used for experiments.

Calu-3 cells were cultured in minimum essential media (MEM, Life Technologies) supplemented with 10% (v/v) FCS, 1% (v/v) penicillin-streptomycin, 1% (v/v) non-essential amino acids solution (NEAA, Life Technologies), and 1% (v/v) sodium pyruvate. To maintain the cells, the culture medium was aspirated, and the cells were washed once with 13 mL phosphate-buffered saline (PBS). Subsequently, 3.5 mL of pre-warmed Trypsin/EDTA was added to the flask, and the cells were incubated at 37°C for 10–20 min, monitoring for detachment. Once detached, the cells were neutralized by adding 6.5 mL fresh medium and gently resuspended by pipetting. The cell suspension was transferred to a 15 mL Falcon tube and centrifuged at 1790 rpm (300 x g) for 5 min at room temperature, followed by aspiration of the supernatant. The cell pellet was resuspended in 5 mL fresh medium, and cell viability was assessed. For routine cultivation, 2.5 x 10^6 cells were seeded in a new T75 flask with a final volume of 13 mL. Cells were fed every 2-3 days and maintained in a humidified incubator at 37°C with 5% CO_2. All reagents for both cells were prewarmed to 37 °C before use.

Transwell Preparation and Transepithelial Electrical Resistance (TEER) Experiments

To conduct Transepithelial Electrical Resistance (TEER) measurements under liquid-covered conditions (LCC), Calu-3 and Arlo cells were seeded separately onto 0.33 cm² permeable cell culture inserts (400 nm pore size; Corning, 3470) at a density of 3 x 10^4 and 3.3 x 10^4 cells per insert, respectively, allowing 8–10 days for cells to establish tight junctions. Apical inserts received 200 µL of this cell suspension, while the basolateral compartments were filled with 800 µL cell culture medium. To prevent evaporation, outer wells were filled with 800 µL phosphate-buffered saline (PBS), and two wells per plate were designated as blank controls, containing cell culture medium only.

Throughout the culture period, medium changes were performed every 2 to 3 days, with fresh MEM (for Calu-3) or SAGM (for Arlo) medium replacing the old medium in both apical and basolateral compartments. On day 8–10, TEER measurements were initiated. In the first step, TEER measurements were taken for each well in the transwell plate. Before measurement, the electrode

was sterilized using 70% isopropanol and then equilibrated in PBS until reaching a TEER value below 20 Ω·cm². The culture medium was then replaced with 200 µL Hanks' balanced salt solution containing $CaCl_2$ and $MgCl_2$ (HBSS, Gibco, Thermo Fisher Scientific Inc.), and cells were incubated at 37°C for 30–60 min. Following incubation, TEER measurements were conducted again using a suitable electrode system and the EVOM2 with STX2 electrode (WPI). Next, HBSS was removed from the apical and basolateral. Supernatants and compounds of interest were added to the apical side to reach the desired concentrations in a final volume of 200 µL, and the basolateral compartments were filled with 800 µL HBSS. In parallel, several controls were included: blank inserts (without cells) containing only HBSS in both compartments to account for background resistance; a medium control, consisting of inserts with cells exposed to HBSS (including the equivalent DMSO concentration used for compound solubilization), representing the negative control; and a sn control, where cells were treated with wt PAO1 sn alone (without compounds), serving as the positive control for barrier disruption. Plates were incubated at 37°C + 5% CO_2 on a plate shaker at 200 rpm. TEER was recorded at 1 h intervals for up to 7 h. Basolateral and apical medium samples, as well as cell samples, were collected post-measurement for further analysis. The blank-corrected mean values of the data were used for calculations, with the unit area resistance (UAR) calculated as follows:

$$UAR\ [\Omega *\ cm^2] = \left(\Omega_{monolayer} - \Omega_{blank}\right) * effective\ membrane\ area$$

where the effective membrane area was 0.33 cm². The initial UAR at t=0 was set to 100%, and subsequent changes in transepithelial electrical resistance were calculated relative to this baseline.

Transport Experiments and P_{app} Calculations

The transport of fluorescein sodium (FluNa) across cellular monolayers was evaluated concurrently with TEER measurements, using hourly sampling. After the cells were incubated with HBSS and a TEER measurement was taken, the apical and basolateral compartments were aspirated, and 176 µL of FluNa (10 µg/mL in HBSS) was added to the apical (donor) compartment of each transwell. Additional 44 µL of specific test solutions was added to each well, while 800 µL of HBSS was added to the basolateral (acceptor) compartment. Immediately after adding these solutions, samples of 20 µL from the apical and 200 µL from the basolateral compartments were taken and transferred to a 96-well plate for measurement of initial concentrations. The transwell plates were then placed on an orbital shaker set to 150 rpm (IKA, Germany) in an incubator at 37°C in a humidified atmosphere with 5% CO_2, and basolateral samples (200 µL) were collected every 60 min over a 7 h period. Each sample removal was followed by replenishing with 200 µL of HBSS. A calibration curve was prepared using a 1:2 dilution of FluNa, and duplicate concentrations (200 µL) were loaded into the first two rows of the plate. At the experiment's conclusion, a final TEER measurement was recorded, and fluorescence was assessed in the 96-well plate with a CLARIOstar plate reader using excitation and emission wavelengths of 488 nm and 530 nm, respectively. The gain was calibrated against the highest concentration in the calibration curve. The apparent permeability (Papp) of FluNa was subsequently calculated based on the following equation:

$$P_{app}\ [cm/s] = \frac{dQ}{dt}\left[\frac{\mu g}{s}\right] \times \frac{1}{A\ [cm^2] \times C_0\ [\frac{\mu g}{mL}]}$$

Sample Collection after TEER

At the conclusion of the TEER experiment, samples were collected from the apical and basolateral compartments, along with the cells, for subsequent analyses, including gene expression studies, CBA, E-cadherin protein quantification via western blot, and lactate dehydrogenase (LDH) assay. The supernatant from the apical compartment was centrifuged at 300x g for 4 min at room temperature. Two 60 µL aliquots of the supernatant were then transferred into separate tubes for LDH and CBA analyses. For gene expression studies, cells were first washed with 200 µL of cold PBS, then incubated with 350 µL of buffer RLT (Qiagen) at room temperature for 5 min. Cells were subsequently detached from the surface using a pipette tip, transferred into fresh tubes, and stored at -80°C for future RNA extraction. Cell lysates used for E-cadherin quantification were prepared following the method outlined by Aljohmani *et al*.[68]

LDH Quantification

LDH release from each sample was measured using the CytoTox 96® Non-Radioactive Cytotoxicity Assay (Promega). All reagents were prepared as per the manufacturer's instructions. A 50 µL portion of the apical samples, previously stored at -80°C, was transferred into a flat-bottom 96-well plate (Thermo Scientific™ Nunc™). An additional 50 µL of substrate mix was then added to each well. The plate was incubated for 30 min at room temperature, and protected from light. Following incubation, 50 µL of stop solution was added to each well, and absorbance was read at 490 nm using the PHERAstar instrument. Background absorbance from blank wells containing HBSS, substrate, and stop solution only was subtracted from all sample readings to correct for baseline signal prior to plotting.

Gene expression analysis in Calu-3 and Arlo cells

RNA was extracted from the cells using the RNeasy Micro Kit (Qiagen) following the manufacturer's instructions, with minor adjustments: centrifugation times were extended from 15 seconds to 30 seconds, and RNA was eluted in 11 µL of RNase-free water. RNA purity and concentration (ng/µL) were assessed using the NanoDrop™ 2000 spectrophotometer (Thermo Fisher Scientific). All RNA samples were then reverse transcribed to cDNA using the High-Capacity cDNA Reverse Transcription Kit (Applied Biosystems™). Target genes were quantified via qPCR using the PowerUp™ SYBR™ Green Master Mix (Applied Biosystems™) on the StepOne Plus instrument, in accordance with the manufacturer's guidelines. Data analysis was performed using the ΔΔCt method, with fold changes calculated relative to the healthy control.

Quantification of *lasB* expression in *P. aeruginosa* strains

Overnight cultures of *P. aeruginosa* strains PAO1, Δ*lasB* PAO1, PA NH 57388A muc., and PA RP73 were grown in LB medium. Cultures were adjusted to an OD_{600} of 1, and cell lysates were prepared by adding two volumes of QIAGEN RNAprotect® Bacteria Reagent. After vortexing and resting at room temperature for 5 min, samples were centrifuged at 5000x g for 10 min at room temperature. Pellets were resuspended in 20 µL Proteinase K combined with 200 µL TE buffer (30 mM Tris-Cl, 1 mM EDTA, pH 8.0, 15 mg/mL lysozyme), vortexed every 2 min over a 10-min period. Following this, 700 µL of buffer RLT (QIAGEN RNeasy Plus Kit) was added, and samples were vortexed and centrifuged with the gDNA eliminator spin column at 14000 rpm for 2 min. The flow-through was combined with 700 µL of 99% ethanol for RNA extraction using the QIAGEN RNeasy Plus Kit, with centrifugation times extended to 2 min. RNA was eluted with RNase-free water and treated with Invitrogen™ DNA-free™ DNA Removal Kit to ensure complete removal of genomic DNA.

Concentrations were measured on a NanoDrop 2000 (Thermo Fisher Scientific), and 100 ng of RNA was reverse transcribed using the High-Capacity cDNA Reverse Transcription Kit (Applied Biosystems™).

For qPCR, TaqMan™ Fast Advanced Master Mix (Applied Biosystems™) was used and the absolute quantification was carried out as described in Kiefer, Schütz *et al*.[46] The qPCR was run on a StepOnePlus™ Real-Time PCR System, with thermal cycling conditions as specified in the TaqMan™ protocol. Data were analyzed using the standard curve method, and quantities of *lasB* in copy number per microliter were calculated for each strain.

Cytometric Bead Array (CBA)

Cytokine levels were quantified using a bead-based fluorescence-activated cell sorting (FACS) assay with the CBA method, utilizing Human Soluble Protein Flex Sets specific for IL-6 (558276), TNF (558273), G-CSF (558326), MCP-1 (558287), and IL-1β (558279), along with the Human Soluble Protein Master Buffer Kit (558264), all purchased from BD Biosciences. The assay followed the manufacturer's protocol, with cytokines quantified against their respective standard curves. Bead sorting and analysis were conducted using a BD LSRFortessa™ FACS (BD Biosciences), and data analysis was performed with FCAP Array Version 3.0.1 (BD Biosciences).

Quantification of E-Cadherin *via* Western blot

The procedure was performed as described by Aljohmani *et al*. with a modification in the lysis buffer volume.[68] Briefly, cultured cells were lysed in 50 µL lysis buffer (20 mM Tris·HCl, 150 mM NaCl, 1% Triton X-100, 1 mM EDTA, 1 mM Na_3VO_4, 1 mM PMSF, 10 mM 1,10-phenanthroline monohydrate) supplemented with 1× Complete Inhibitor (Roche Diagnostics Deutschland GmbH, Mannheim, Germany) and incubated at 4°C for 10 min. Cell lysates were centrifuged at 16,000 g for 15 min at 4°C, and the supernatant was used for protein quantification via a commercial Bicinchoninic acid assay (BCA) kit (Thermo Fisher, Karlsruhe, Germany), following the manufacturer's instructions. The subsequent steps, including SDS-PAGE, protein transfer, blocking, antibody incubation, chemiluminescence detection, and densitometric quantification, were performed as detailed in Aljohmani *et al*.

Visualization of E-Cadherin and Claudin-4 *via* Confocal Laser Scanning Microscopy (CLSM)

Arlo and Calu-3 cells were seeded into 24-well transwell inserts and cultured for 10 days under the same conditions used for the TEER experiments. After incubation, the basolateral and apical fluids were aspirated, and both compartments were washed three times with PBS. To fix the cells, 200 µL of 4% paraformaldehyde (Thermo Fisher Scientific Inc., Netherlands) in PBS was added to the apical chambers for 15 min at room temperature. After fixation, the apical chambers were washed three times with PBS. For staining, the transwell inserts were transferred into a new 24-well plate, and the PBS was removed. Both the apical and basolateral chambers were washed twice with HBSS. To permeabilize the cells, a permeabilization buffer consisting of 1% BSA and 0.05% saponin in PBS was added to both chambers, and the cells were incubated at room temperature for 1 h on a shaker plate (MTS 2/4 D S1 Microplate Shaker, IKA, Germany) at 150 rpm. All staining steps were performed in the dark. After the permeabilization step, the buffer was aspirated, and the cells were incubated overnight at 4°C with 200 µL of a primary antibody mixture containing mouse anti-E-Cadherin IgG (1:50, BD Biosciences, USA) and rabbit anti-Claudin-4 IgG (1:100, Invitrogen, USA) in permeabilization buffer. The following day, the primary antibody solution was removed, and the apical compartments were washed three times with permeabilization buffer, keeping the plate on the shaker

plate at 150 rpm for 10 min per wash. Subsequently, 200 µL of secondary antibodies were added to the apical chambers: anti-mouse goat IgG Alexa Fluor 546 (1:500, Invitrogen, USA) and anti-rabbit goat IgG Alexa Fluor 488 (1:1000, Invitrogen, USA), both in permeabilization buffer, and the cells were incubated for 1 h at room temperature. Afterward, the cells were washed three times with permeabilization buffer under the same conditions as before. For the final staining step, the cells were incubated for 30 min at room temperature with 100 µL of 1 µg/mL DAPI (Sigma Aldrich, Switzerland) in PBS. After incubation, the apical chambers were washed three times with PBS under the same conditions. The cell-covered membranes were then mounted onto 24x50 mm microscopy slides (Thermo Fisher Scientific, Netherlands) using Fluorescence Mounting Medium (DAKO Schweiz AG, Switzerland). A cover slip (Carl Roth, Germany) was placed over the membranes, and the samples were wrapped in aluminum foil and left to dry overnight at room temperature.

For imaging, a Leica TC SP8 Confocal Microscope (Leica Microsystems, Wetzlar, Germany) equipped with a 25x water objective (Fluotar VISIR 25x/0.95 WATER) was used. For Calu-3 cell imaging, the argon laser intensity was set to 30%. DAPI was excited at 405 nm with 10% laser intensity, and its emission was detected in the range of 410–501 nm. Alexa Fluor 488, used to detect Claudin-4, was excited at 488 nm with 40% laser intensity, and the emission was detected from 501 to 561 nm. Alexa Fluor 546, used to detect E-Cadherin, was excited at 561 nm with 3% laser intensity, and its emission was detected from 566 to 701 nm. All signals were captured sequentially in three scans to minimize crosstalk, using PMT detectors for DAPI and Alexa Fluor 546 and a HyD detector for Alexa Fluor 488, with a gain of 800 for the PMT detectors and 100 for the HyD detector. For Arlo cell imaging, the argon laser intensity was also set to 30%. DAPI was excited at 405 nm with 30% laser intensity, and its emission was detected in the range of 410–494 nm. Alexa Fluor 488, used to detect Claudin-4, was excited at 488 nm with 70% laser intensity, and its emission was detected from 494 to 564 nm. Alexa Fluor 546, used to detect E-Cadherin, was excited at 561 nm with 6% laser intensity, and its emission was detected from 566 to 701 nm. Signals were captured sequentially in three scans to minimize crosstalk, with PMT detectors for DAPI and Alexa Fluor 546 and a HyD detector for Alexa Fluor 488, with a gain of 800 for the PMT detectors and 100 for the HyD detector. Images were captured at a resolution of 2048 x 2048 with bidirectional X scanning at a scan speed of 100 Hz. Line averaging and frame accumulation of 2 were applied, and a 3x virtual zoom was used for obtaining close-up pictures. Further image editing and compilation was done by using the open-source software ImageJ and GIMP.

RNA Sequencing

Calu-3 cells were exposed to various treatment conditions, including supernatants from wild-type (wt) *P. aeruginosa* (wt PAO1 sn and Δ*lasB* PAO1 sn), 100 µM compound **1** combined with wt PAO1 sn, as well as an HBSS control, an off-target control with compound **1** + Δ*lasB* PAO1 sn, and a compound toxicity control. Following a 7 h incubation, total RNA was extracted using Buffer RLT (Qiagen), and further purified with the Monarch® RNA Cleanup Kit (10 µg capacity, New England Biolabs). RNA integrity and purity were assessed, with all samples showing RNA Integrity Numbers (RIN) greater than 8.

Strand-specific mRNA sequencing was performed. mRNA libraries were prepared using the NEBNext® Ultra™ II Directional RNA Library Prep Kit (NEB #E7765), followed by sequencing on an Illumina NovaSeq 6000 platform using paired-end 50 bp reads (PE50, 2 × 50 bp), yielding approximately 30 million reads per sample and a total flow cell output of 800 million clusters. Raw FASTQ files were analyzed using a standardized pipeline deployed in a Docker container running RNADetector.[69] STAR was employed for alignment, and gene-level quantification was performed

using *feature*Counts. Read count matrices were normalized to correct for library size and composition biases using the Bioconductor package edgeR. Pathway enrichment analyses were conducted using the Reactome pathway knowledgebase. The count matrix derived from RNAdetector was modified to only contain identifiers, and the matrix was analyzed using the PADOG analysis method, which includes disease pathways, without any additional normalization. Input matrix and data of the resulting PDF, R-Script and Excel report files are provided in Data S1.

Gene expression profiles were visualized through volcano plots and heatmaps generated in Python (v3.x) using numpy (v2.0.2), pandas (v2.2.3), matplotlib (v3.9.4), and seaborn (v0.13.2). Visualizations were based on a normalized count table and the list of significantly regulated genes identified by RNA-detector (see lists in Data S1). Selected genes showing significant differential expression influenced by LasB activity and its inhibition by compound **1** were further validated by quantitative PCR following the aforementioned protocol.

Calu-3 transwell-based bacterial infection model

Wild type PAO1 and PAO1 Δ*lasB* strains were cultured overnight in LB medium at 37°C with shaking at 180 rpm. The following day, cultures were centrifuged (5000 rpm, 4°C, 10 min), resuspended in fresh LB, and adjusted to an OD_{600} of 0.025. Cultures were then grown to an OD_{600} of approximately 2, centrifuged again, and standardized to an OD_{600} of 1.5. A 1:10 dilution was performed to achieve the desired MOI of 30 for the experiment.

Calu-3 cells were seeded in transwells and grown for 9 days as described above. On the day of the experiment, TEER was measured, and cells were washed with HBSS. After a 30–60 min incubation in HBSS at 37°C + 5% CO_2, TEER was measured again. The apical compartment received 190 µL HBSS with 10 µL of diluted bacterial culture. For compound testing, 1 µL of compound **3** (in DMSO) or meropenem (in PBS) was added, maintaining a final DMSO concentration of 0.5%. The basolateral compartment received 800 µL HBSS. Plates were incubated at 37°C with 5% CO_2, and TEER was measured hourly for 7 h.

To quantify bacterial growth, 10 µL samples were collected from the apical compartment at the start and end of the experiment. These samples were serially diluted, plated on agar, and incubated. Colony-forming units (CFUs) were counted the following day.

Determination of Meropenem Minimum Inhibitory Concentration (MIC) Against *Pseudomonas aeruginosa* PAO1

The antibacterial activity of meropenem was determined in *Pseudomonas aeruginosa* PAO1 (DSM 19880). As a bacteria start OD_{600}, we used 0.03 (optical density of the bacteria at 600 nm) in a total volume of 200 µL in 99.5% HBSS and 0.5% lysogeny broth (LB) medium containing meropenem pre-dissolved in DMSO (maximal DMSO concentration: 1%). Final meropenem concentrations prepared from serial dilutions ranged from 0.0625 to 5 µM. The OD_{600} values were determined directly after addition of the antibiotic and again after incubation at 37°C for 16 h and 50 rpm in 96 well plates (Sarstedt, Nümbrecht, Germany) using a FLUOstar Omega microplate reader (BMG labtech, Ortenberg, Germany). The given MIC value is a mean of two independent determinations and is defined as the lowest concentration of meropenem that reduced the OD_{600} by ≥ 95%.

Statistical analysis and data visualization

All graphical data are presented as mean ± standard deviation (SD). Statistical comparisons between experimental conditions were performed using Dunnett's one-way ANOVA to determine significant

differences. A p-value ≤ 0.05 was considered statistically significant, while p-values > 0.05 were considered non-significant. Graphs were generated using GraphPad Prism (v 9.3.1). Transcriptomic data were analyzed and visualized as heatmaps and volcano plots *via* Python (v3.x).

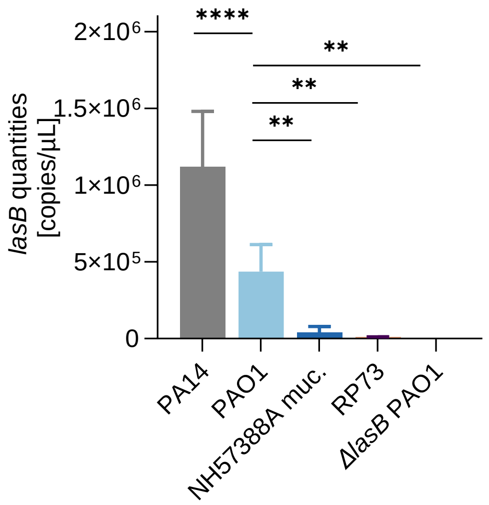

Figure S1: Absolute quantification of *lasB* expression across various *Pseudomonas aeruginosa* strains. *lasB* transcript levels were measured in PAO1, PA14, NH57388A muc., RP73, and Δ*lasB* PAO1 using a standard curve-based absolute quantification approach. PA14 showed the highest expression, followed by PAO1, while NH57388A muc. and RP73 exhibited minimal to undetectable levels. Data represent mean values from three independent experiments ($n = 3$); error bars indicate standard deviation (SD). Statistical analysis was performed using one-way ANOVA with Dunnett's multiple comparisons test, comparing each strain to PAO1. (****$p \leq 0.0001$; **$p \leq 0.01$).

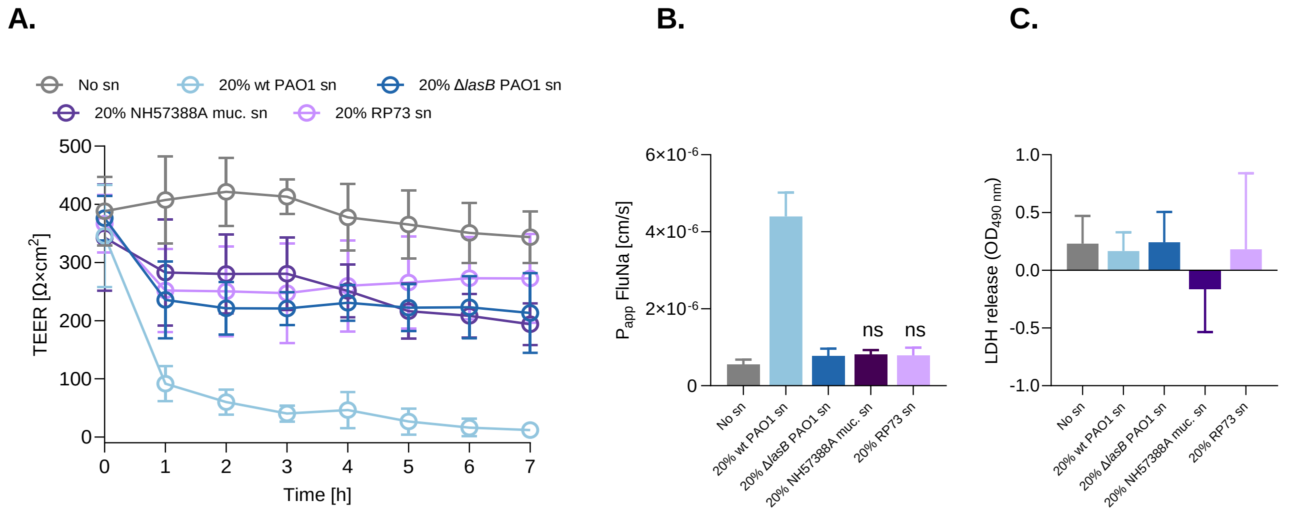

Figure S2: Effect of *Pseudomonas aeruginosa* strains on epithelial barrier integrity. (A) Transepithelial electrical resistance (TEER) and **(B)** fluorescein sodium (FluNa) permeability (P_{app}) measured in Calu-3 cells over a 7-hour period following treatment with 20% (v/v) supernatants (sn) from wild-type (wt) PAO1, Δ*lasB* PAO1, NH57388A muc., and RP73 strains. **(C)** Lactate dehydrogenase (LDH) release assessed at 7 hours to evaluate cytotoxicity. Data represent mean ± standard deviation (n = 3 independent experiments). Statistical analysis was conducted using one-way ANOVA followed by Dunnett's multiple comparisons test, comparing each condition to Δ*lasB* PAO1 sn (****$p \leq 0.0001$; ns, not significant).

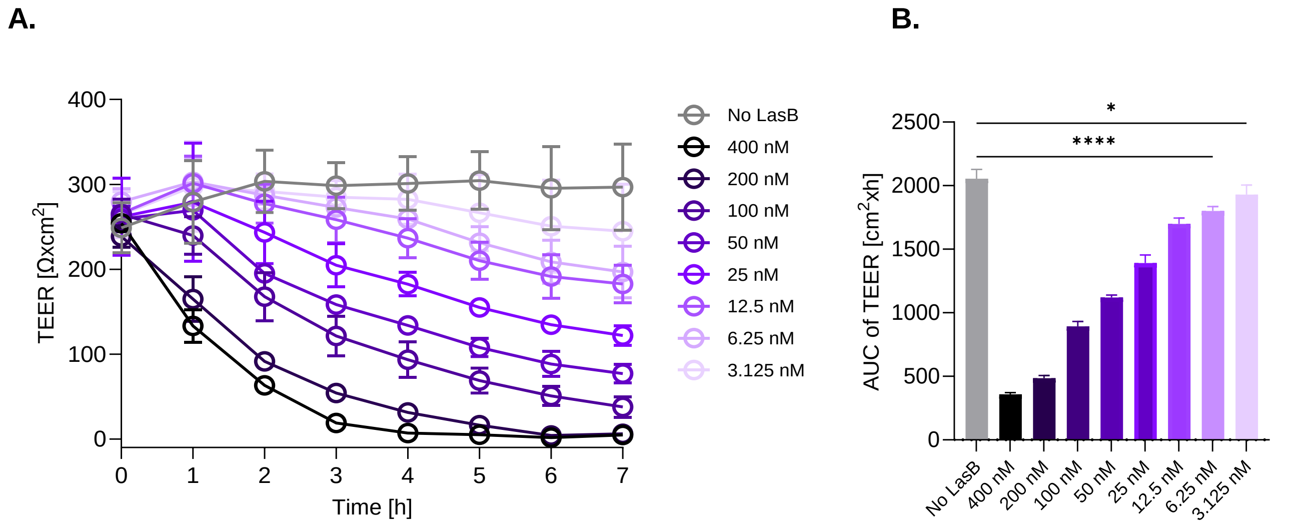

Figure S3: Effect of purified LasB of *Pseudomonas aeruginosa* on Calu-3 epithelial barrier integrity. (A) Transepithelial electrical resistance (TEER) of Calu-3 monolayers following treatment with increasing concentrations of purified LasB protein, monitored over a 7-hour period. **(B)** Area under the curve (AUC) analysis of TEER data quantifies the dose-dependent disruption of barrier function. Data represent mean ± standard deviation (n = 2 independent experiments). Statistical analysis was performed using one-way ANOVA followed by Dunnett's multiple comparisons test against the healthy control (****$p \leq 0.0001$; *$p \leq 0.05$; ns, not significant).

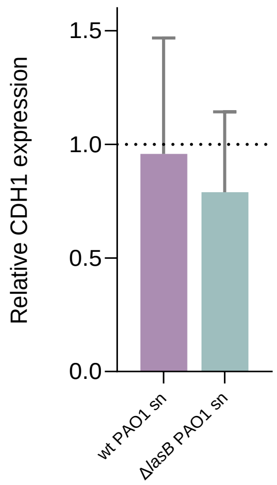

Figure S4: CDH1 gene expression in Calu-3 cells exposed to *Pseudomonas aeruginosa* supernatants. Relative expression of CDH1 in Calu-3 cells following exposure to 20% (v/v) wild-type (wt) PAO1 supernatant (sn) or Δ*lasB* PAO1 sn relative to the unchallenged healthy control.

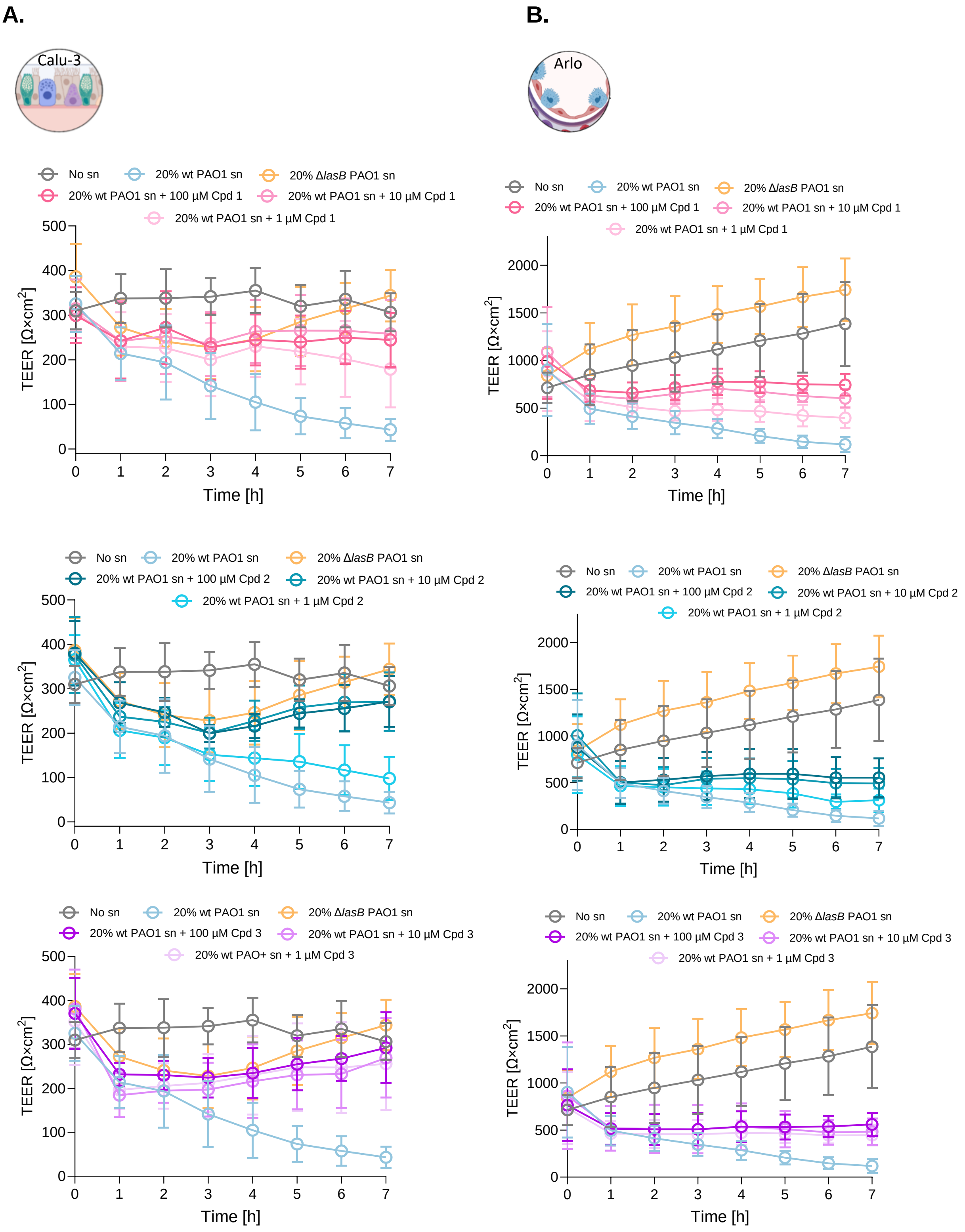

Figure S5: Evaluation of LasB inhibitors in preserving epithelial barrier integrity. Transepithelial electrical resistance (TEER) of **(A)** Calu-3 and **(B)** Arlo cells over 7 hours following exposure to 20% (v/v) wild-type (wt) *Pseudomonas aeruginosa* PAO1 supernatant (sn) or Δ*lasB* PAO1 sn, with or without treatment using LasB inhibitors at 1, 10, or 100 µM. Data represent mean ± standard deviation (n = 3 independent experiments).

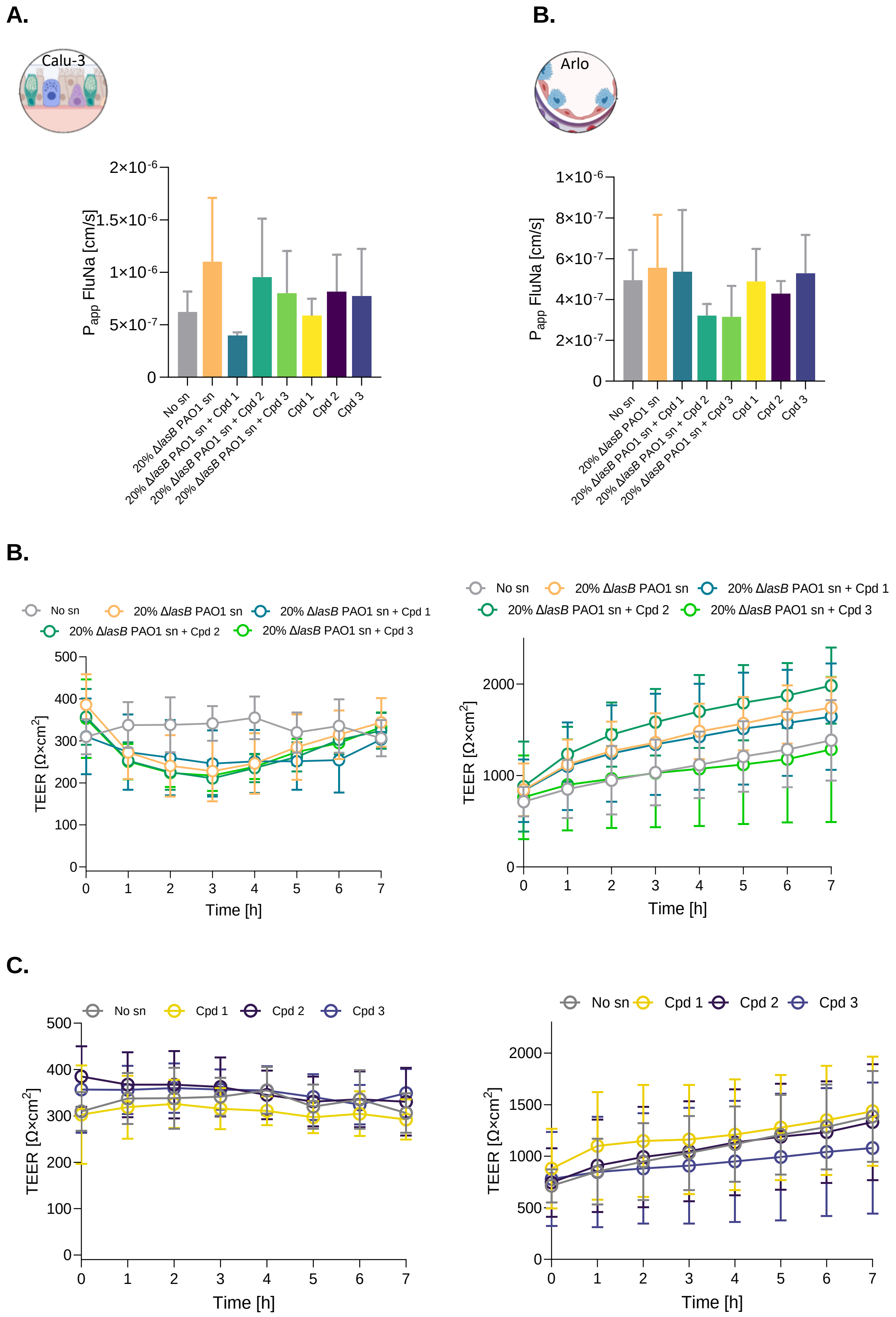

Figure S6: Evaluation of off-target effects and cytotoxicity of LasB inhibitors in Calu-3 and Arlo cells. **(A)** Paracellular permeability (P_{app}) of Calu-3 and Arlo cells following treatment with compounds alone or in combination with Δ*lasB* *Pseudomonas aeruginosa* PAO1 supernatant (sn). **(B)** Transepithelial electrical resistance (TEER) measurements over 7 hours in both cell lines treated with compounds and Δ*lasB* PAO1 sn to assess potential off-target effects. **(C)** TEER over 7 hours following treatment with compounds alone to

evaluate cytotoxicity. Data represent mean ± standard deviation (n = 3 independent experiments). Statistical analysis was performed using ordinary one-way ANOVA followed by Dunnett's multiple comparisons test, comparing all treatment groups to the healthy control (no sn); no statistically significant differences were observed.

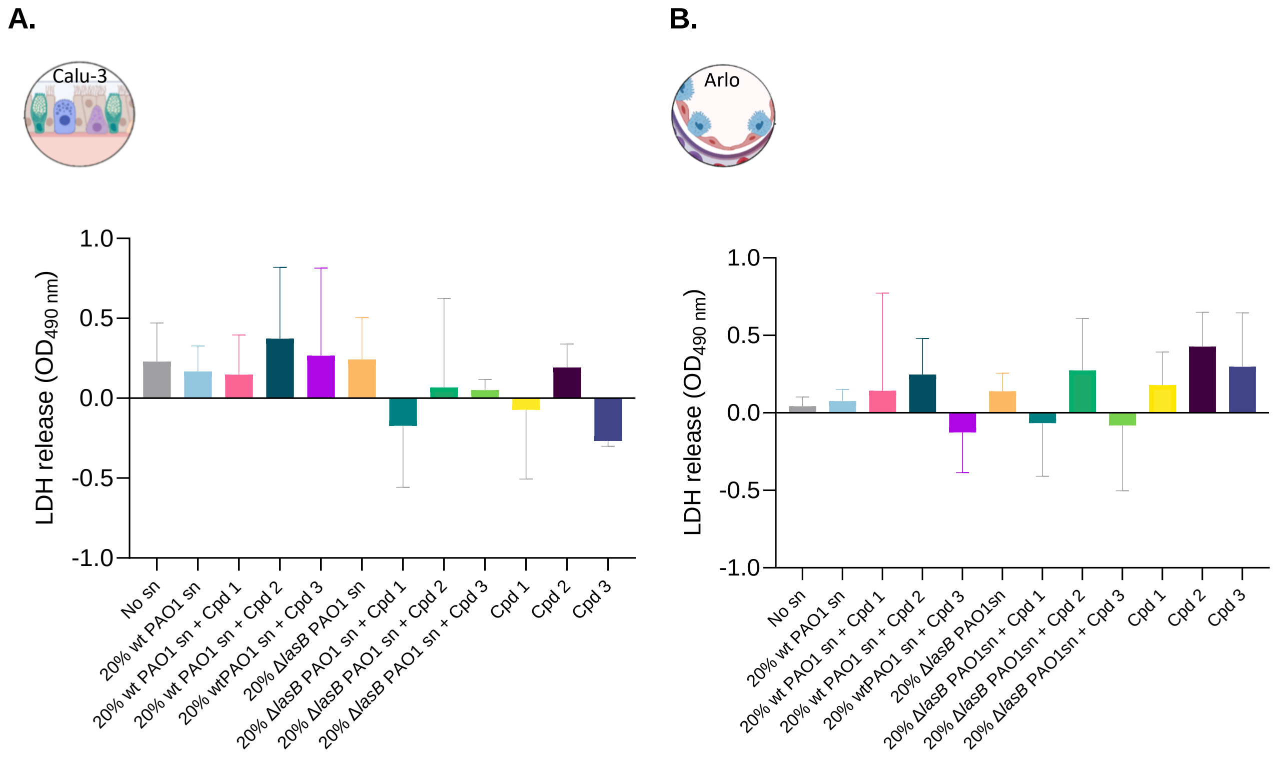

Figure S7: Lactate dehydrogenase (LDH) release in Calu-3 and Arlo cells. LDH release was measured in **(A)** Calu-3 cells and **(B)** Arlo cells under various experimental conditions with, without *Pseudomonas aeruginosa* PAO1 supernatant and with, without compounds. LDH levels were quantified relative to the healthy control no supernatant (no sn). Data represent mean ± standard deviation (n = 3 independent experiments). Statistical analysis was performed using ordinary one-way ANOVA followed by Dunnett's multiple comparisons test, comparing each treatment group to the healthy control. No statistically significant differences were observed in any experimental condition.

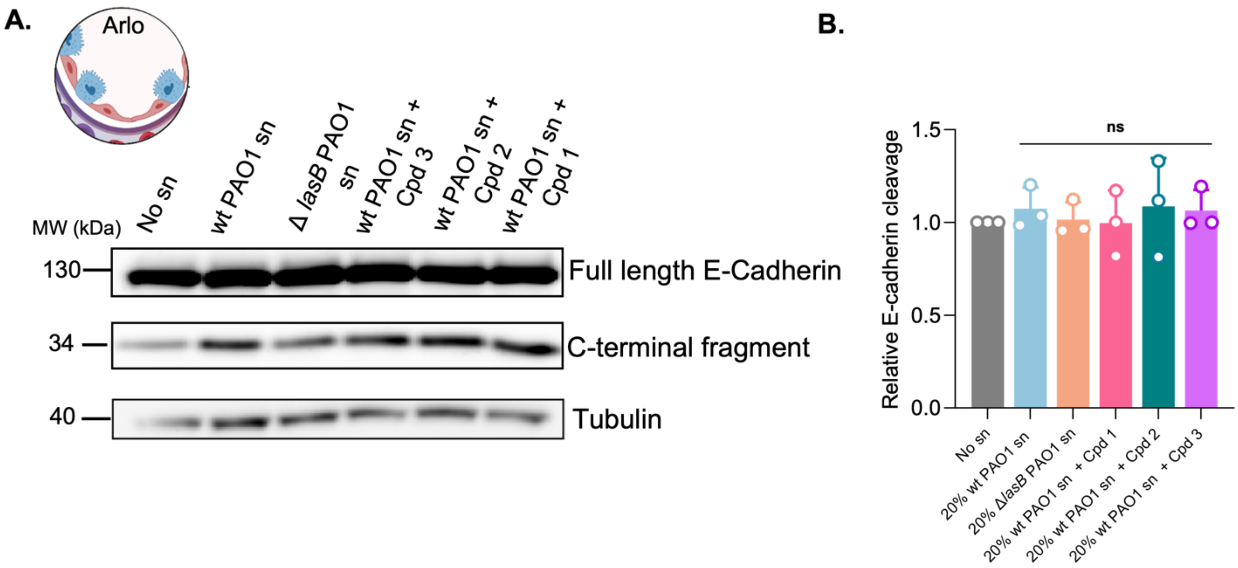

Figure S8: Quantification of E-Cadherin in Arlo cells upon challenge with *Pseudomonas aeruginosa* PAO1 supernatant, with or without inhibitor. (A) Western blot analysis of E-cadherin cleavage in Arlo cells under healthy conditions or following challenge with wild-type (wt) *Pseudomonas aeruginosa* PAO1 supernatant (sn), Δ*lasB* PAO1 sn, or wt PAO1 sn with 100 µM of LasB inhibitors (**1–3**). Full-length and C-terminal fragment bands are shown, with tubulin as a loading control. **(B)** Relative quantification of E-cadherin cleavage in Calu-3 cells under the same experimental conditions. Data represent mean ± standard deviation (n = 3 independent experiments). Statistical analysis was conducted using one-way ANOVA followed by Dunnett's multiple comparisons test. (ns, not significant).

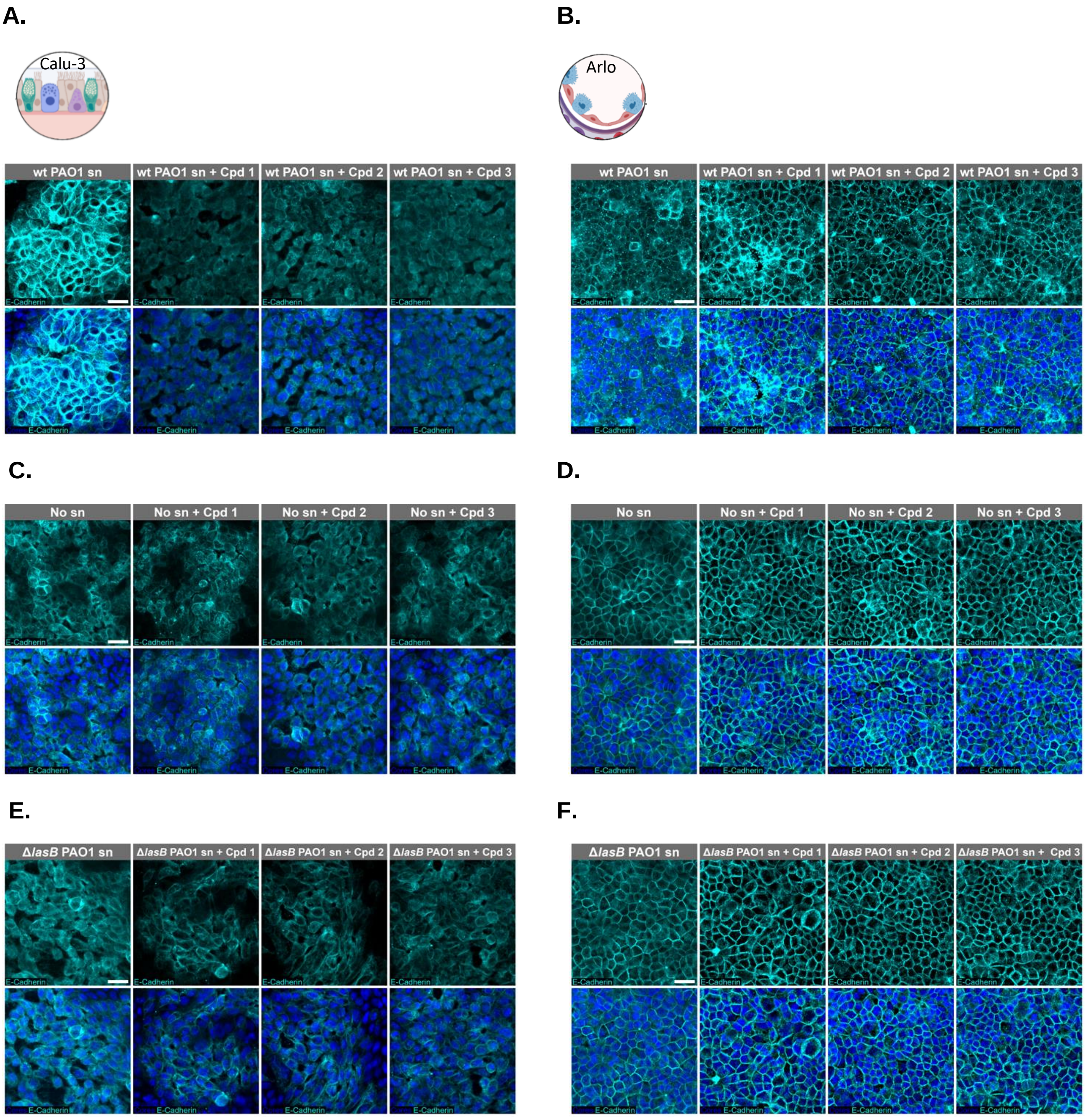

Figure S9: Imaging of E-cadherin (cyan) localization in Calu-3 (left) and Arlo cells (right) under different treatment conditions through confocal laser scanning microscopy (CLSM). **(A)** Cells subjected to 20% (v/v) wild-type (wt) *Pseudomonas aeruginosa* PAO1 supernatant (sn) and visualization of the protective effect of the three different LasB Inhibitors at 100 µM. **(B)** Cytotoxicity tests using no sn on cells as well as applying each compound at 100 µM. **(C)** Test for off-target side effects through exposure to 20% (v/v) Δ*lasB* PAO1 sn and treatment with 100 µM of the LasB inhibitors. Nuclei were counterstained with DAPI (blue). The scale bar = 25 µm.

A.

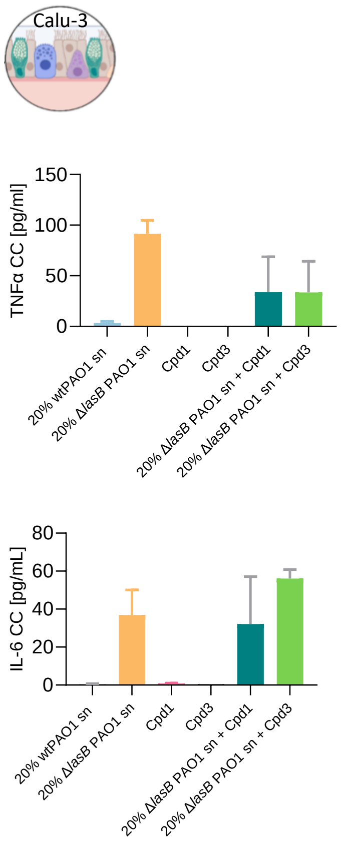
Calu-3
TNFα CC [pg/ml]
IL-6 CC [pg/mL]
20% wtPAO1 sn
20% ΔlasB PAO1 sn
Cpd1
Cpd3
20% ΔlasB PAO1 sn + Cpd1
20% ΔlasB PAO1 sn + Cpd3

B.

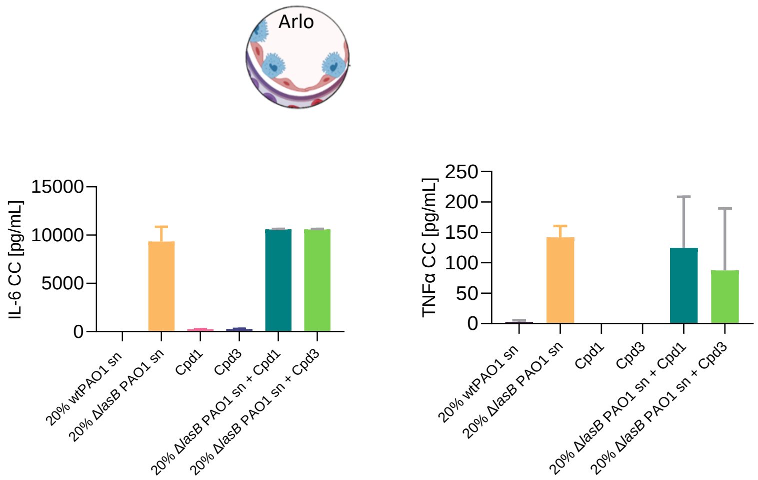
Arlo
IL-6 CC [pg/mL]
TNFα CC [pg/mL]
20% wtPAO1 sn
20% ΔlasB PAO1 sn
Cpd1
Cpd3
20% ΔlasB PAO1 sn + Cpd1
20% ΔlasB PAO1 sn + Cpd3

C.

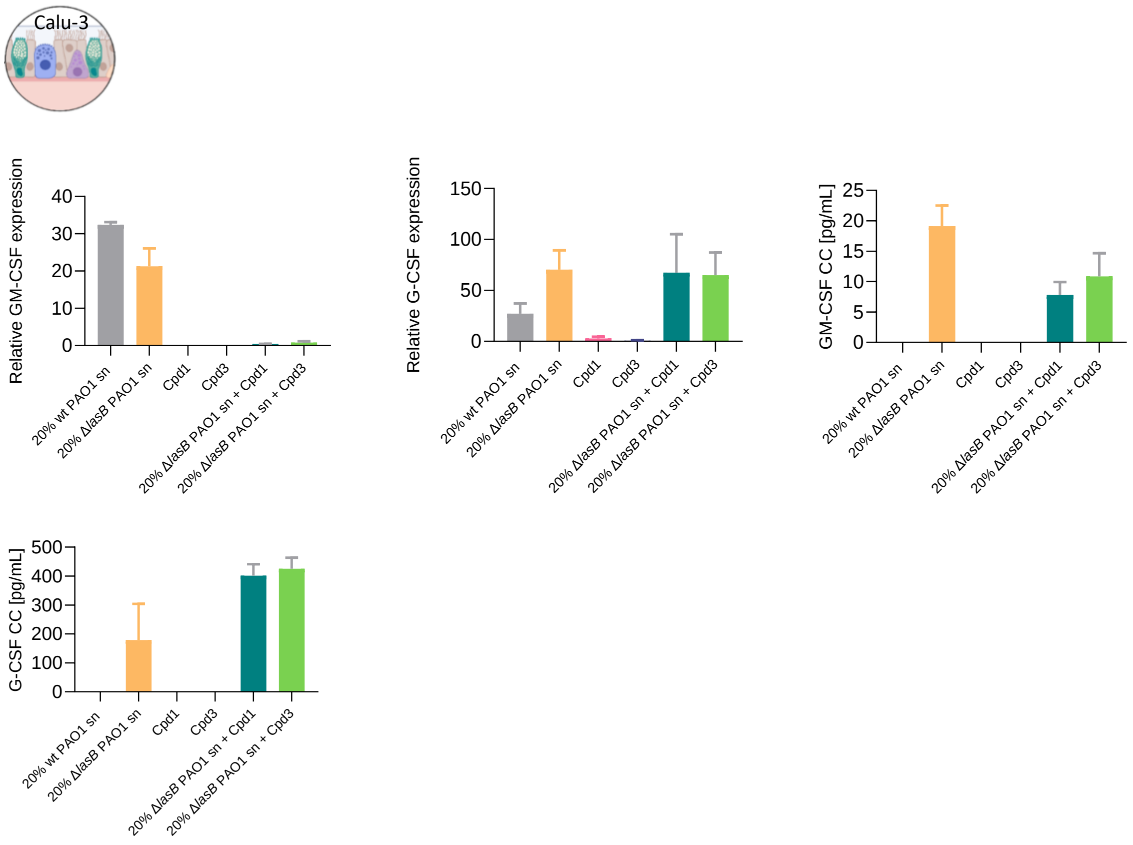
Calu-3
Relative GM-CSF expression
Relative G-CSF expression
GM-CSF CC [pg/mL]
G-CSF CC [pg/mL]
20% wt PAO1 sn
20% ΔlasB PAO1 sn
Cpd1
Cpd3
20% ΔlasB PAO1 sn + Cpd1
20% ΔlasB PAO1 sn + Cpd3

Figure S10: Cytotoxicity, off-target effects, and cytokine profiles of LasB inhibitors. Cytometric bead array (CBA) quantification of TNF and IL-6 in **(A)** Calu-3 and **(B)** Arlo cells. **(C)** Expression and protein analysis of colony-stimulating factors under various treatment conditions. All experiments were conducted using 20% (v/v) wild-type (wt) *Pseudomonas aeruginosa* PAO1 supernatant (sn), Δ*lasB* PAO1 sn, inhibitors alone, or inhibitors combined with Δ*lasB* PAO1 sn. Data represent mean ± standard deviation (*n* = 3 independent experiments).

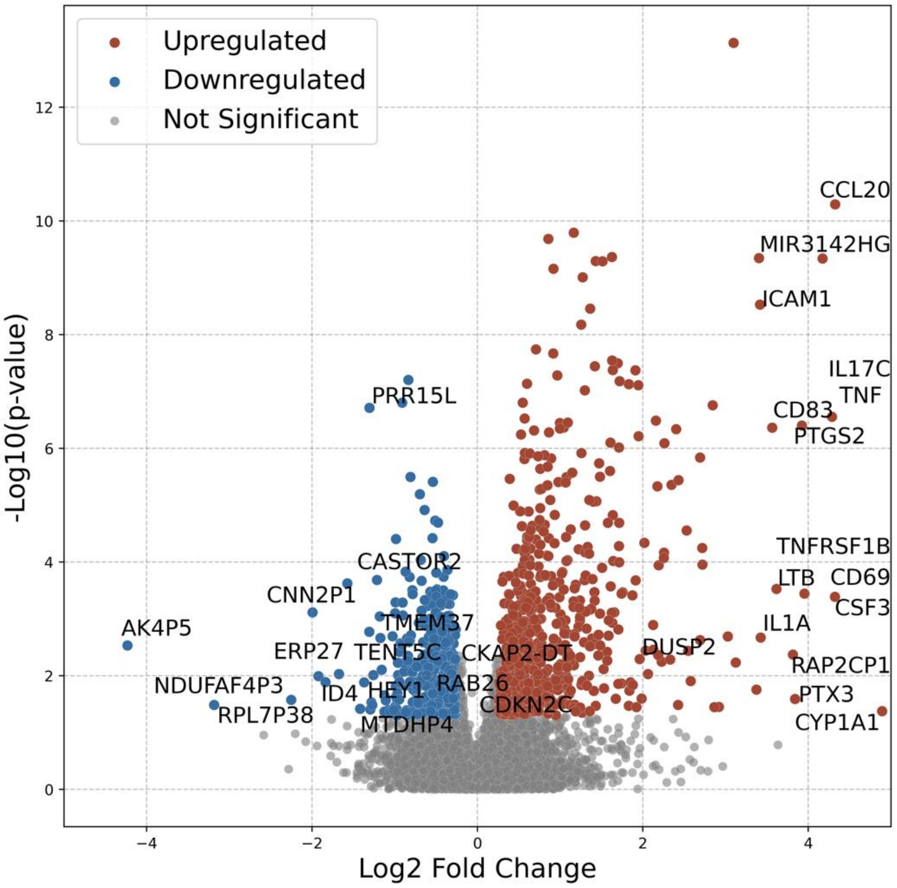

Figure S11: Volcano plot showing the expression of LasB-specific genes identified in Calu-3 cells exposed to LasB-containing *Pseudomonas aeruginosa* supernatant and in NuLi-1 cells infected with *P. aeruginosa* (GSE199424, 8 h). The plot displays $\log_2$ fold changes versus $-\log_{10}$ adjusted p-values. Commonly downregulated and upregulated genes, including *DUSP2*, are highlighted, indicating conserved transcriptional responses to *P. aeruginosa* across epithelial cell types.

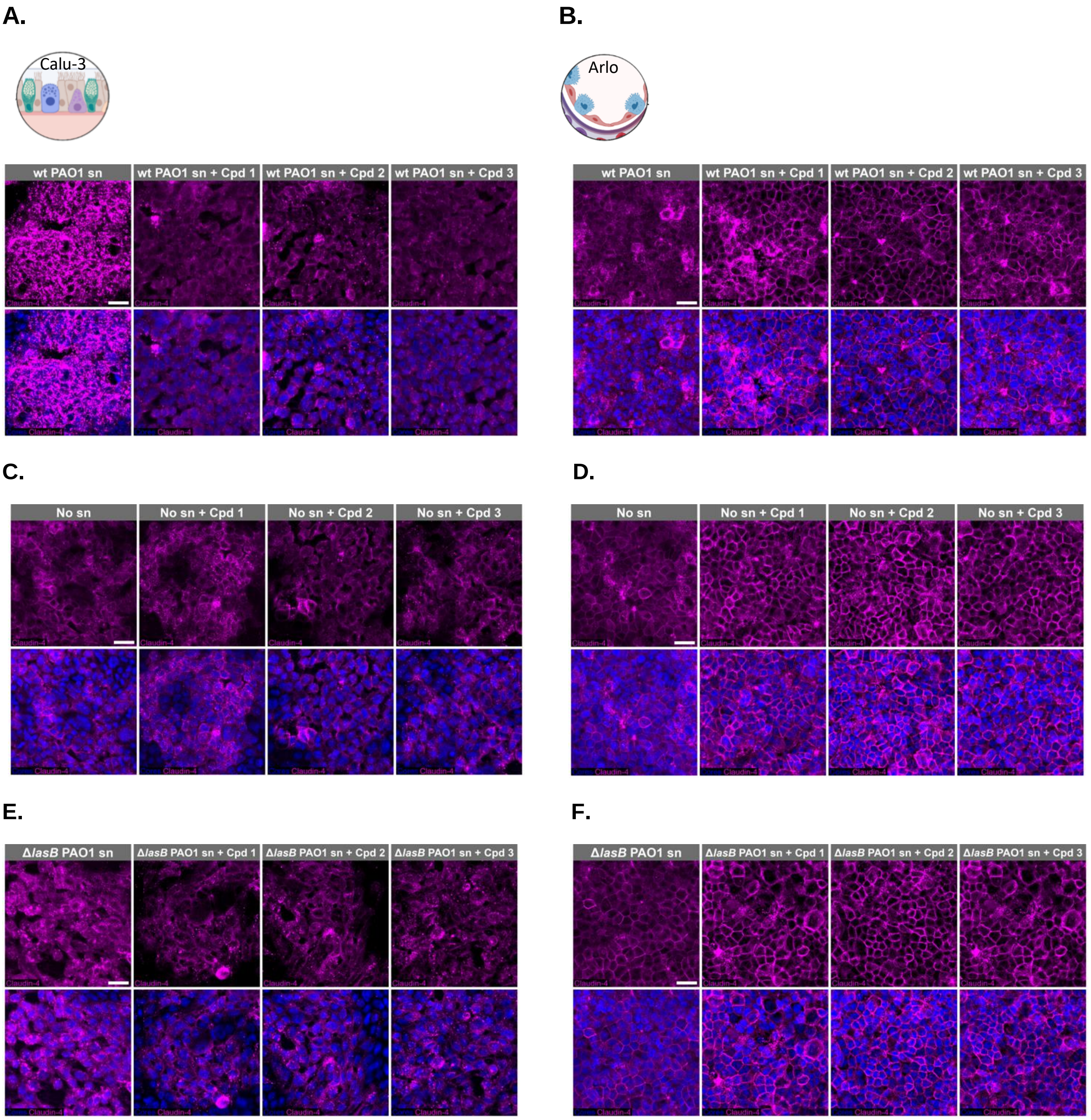

Figure S12: Visualization of Claudin-4 (magenta) localization in Calu-3 (left) and Arlo cells (right) under different treatment conditions through confocal laser scanning microscopy (CLSM). (A) and **(B)** Cells exposed to 20% (v/v) wild-type (wt) *Pseudomonas aeruginosa* PAO1 supernatant (sn), showing the effect of the three different LasB inhibitors at 100 µM. **(C)** and **(D)** Cytotoxicity tests using no sn on cells, as well as applying each compound at 100 µM. **(E)** and **(F)** Test for off-target side effects through exposure to 20% (v/v) Δ*lasB* PAO1 sn and treatment with 100 µM of LasB inhibitors. Nuclei were counterstained with DAPI (blue). Scale bar = 25 µm

A.

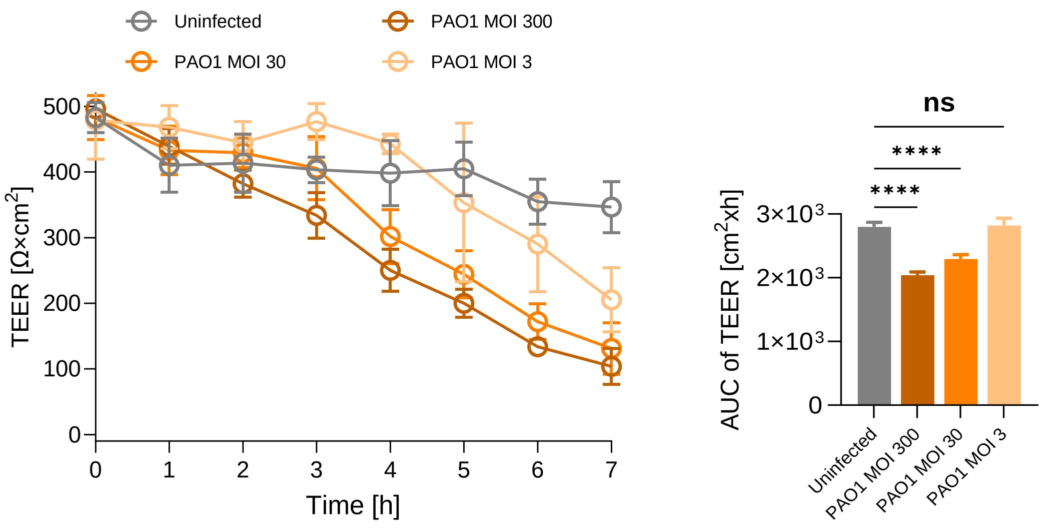

B.

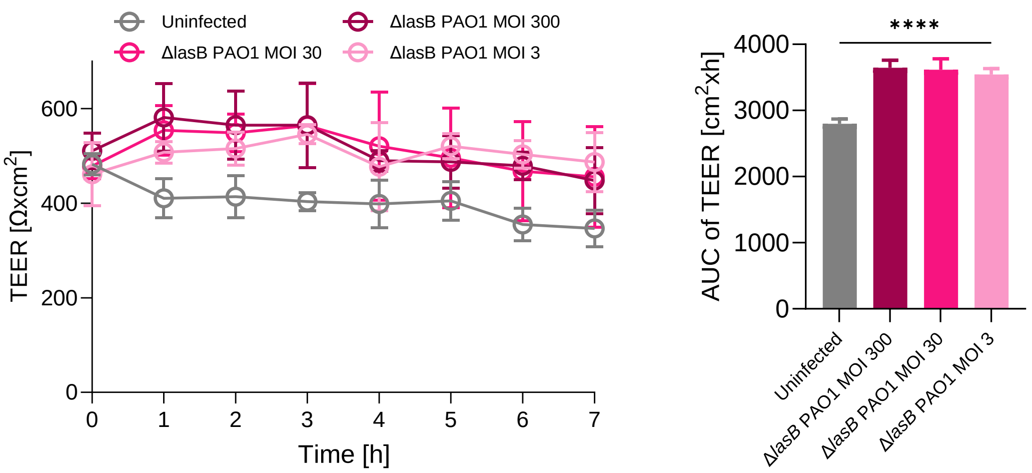

Figure S13: Infection studies of wild-type (wt) and Δ*lasB Pseudomonas aeruginosa* PAO1 on Calu-3 cells. Transepithelial electrical resistance (TEER) of Calu-3 monolayers monitored over 7 hours following infection with **(A)** wt PAO1 and **(B)** Δ*lasB* PAO1 at varying multiplicities of infection (MOIs). Area under the curve (AUC) of TEER data was calculated to assess barrier disruption. Data represent mean ± standard deviation (n = 3 independent experiments). Statistical analysis was performed using ordinary one-way ANOVA followed by Dunnett's multiple comparisons test (****$p \leq 0.0001$; ns, not significant).

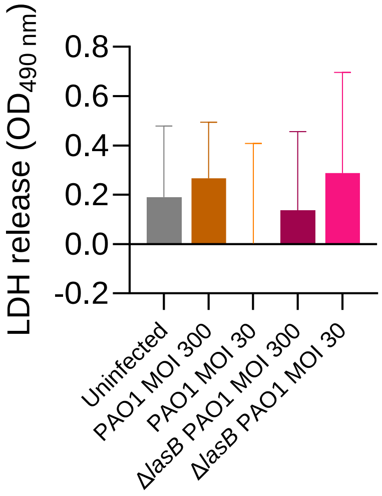

Figure S14: Lactate dehydrogenase (LDH) release in Calu-3 cells upon infection with PAO1. LDH release was measured in Calu-3 cells following infection with wild-type (wt) *Pseudomonas aeruginosa* PAO1 and Δ*lasB* PAO1 at multiplicities of infection (MOI) of 30 and 300. LDH levels were quantified relative to the uninfected control. Data represent mean ± standard deviation (*n* = 3 independent experiments). Statistical analysis was performed using ordinary one-way ANOVA followed by Dunnett's multiple comparisons test, comparing each group to the uninfected control. No statistically significant differences were observed.

Table S1: Chemical structure of selected compounds for this study.

Compound	Chemical structure
**1**	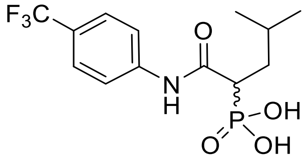
**2**	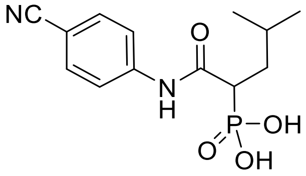
**3**	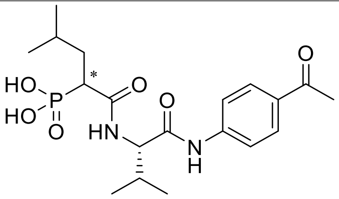

Table S2: List of primers designed for the quantification of cytokine gene expression *via* RT-qPCR.

Gene	Primer	Sequence 5'—3'
POLR2A	Forward	CAGCAAGGTCGTCCTCCCCTGT
	Reverse	GATGCGCAATGGCTTGGTTGAA
GUSB	Forward	CGCCCTGCCTATCTGTATTC
	Reverse	TCCCCACAGGGAGTGTGTAG
IL6	Forward	AGAGGCACTGGCAGAAAACA
	Reverse	TCACCAGGCAAGTCTCCTCA
IL1A	Forward	CAGTGCTGCTGAAGGAGATGC
	Reverse	TGGATGGGCAACTGATGTGAA
IL1B	Forward	TACCCAAAGAAGAAGATGGAA
	Reverse	GAGGTGCTGATGTACCAGTTG
CXCL8	Forward	TCAGAGACAGCAGAGCACAC
	Reverse	CTTGGCAAAACTGCACCTTCA
TNF	Forward	TAGCCCATGTTGTAGCAAACCC
	Reverse	AGGAGGTTGACCTTGGTCTG
CSF3	Forward	GTCCCACCTTGGACACACTG
	Reverse	TTCCCAGTTCTTCCATCTGCTG
CSF2	Forward	CTGGAGCTGTACAAGCAGGG
	Reverse	CACAGGAAGTTTCCGGGGTT
IL4	Forward	CTGTGCTCCGGCAGTTCTAC
	Reverse	TCACAGGACAGGAATTCAAGC
IL13	Forward	GTCTCAGCTGGGCAGTTTTC
	Reverse	GAACTGTCCCTCGCGAAAAAG
IL37	Forward	TGGGGGTCTCTAAAGGGGAG
	Reverse	CAGCTTCATCAGTTTCTCCTTCTTC
IL1RN	Forward	TGCAAGCCTTCAGAATCTGGG
	Reverse	GAGCATGAGGCTCAATGGGT
TGFB1	Forward	GGAAATTGAGGGCTTTCGCC
	Reverse	CCGGTAGTGAACCCGTTGAT
CCL2	Forward	TCTCGCCTCCAGCATGAAAG
	Reverse	GGCATTGATTGCATCTGGCT
IRF7	Forward	CGGCTGGAAAACCAACTTCC
	Reverse	GCCTGGGCCTTCTCGC

Table S3: List of primers designed for validation of RNA-seq data by RT-qPCR.

Gene	Primer	Sequence 5'—3'
VIPR1	Forward	TTCTCCTCCATTCAAGGCCG
	Reverse	AACTCGCTGCCTTGTCATCC
DUSP2	Forward	TCCTGTCTACGACCAGGGTG
	Reverse	CCACCATCTGGTTGTCCTCC
SPIRE2	Forward	TCTTCTGCAAGAGAGCCGTC
	Reverse	TGGCAGCTGATACCCTCTGA
CLDN4	Forward	TATTGGGGAGGGACGGAAGT
	Reverse	CCTACCCGGAACAGAGGAGA
CLDN10	Forward	TCATACTGTCAGGGCTGTGC
	Reverse	GGCTCCTGCCCATCCAATAA
FGFBP1	Forward	CGTGTGCTCAGAACAAGGTGAA
	Reverse	CACCTGAGCAGCCAGTAGG
MYD88	Forward	CCTCTCTCCAGGTGCCCAT
	Reverse	GTCTTCAGGGCAGGGACAAG
TCF7	Forward	GCGGGACAACTACGGGAAG
	Reverse	ACCGAATGCATTTCTTTTTCCTC
ACTN4	Forward	GGGAAGCCCTGGAGAAAACA
	Reverse	AGGCCCTCAATCTCCTCGAT
TRIP6	Forward	CACCCTGGAGAAATGTGCCA
	Reverse	TGGGGCAAACTTCCTGTGAA
TM4SF1	Forward	ATGCCTCCGAAAACCACCTC
	Reverse	CTGGCAGGAGCATCAGCAG
BCL6	Forward	GCCTGAGAACCTTGACCTCC
	Reverse	AGCCCGTCATGGACCTGTTA

References

[17] Carius P, Jungmann A, Bechtel M, *et al.* A Monoclonal Human Alveolar Epithelial Cell Line ("Arlo") with Pronounced Barrier Function for Studying Drug Permeability and Viral Infections. Advanced science 2023; 10(8): e2207301 [https://doi.org/10.1002/advs.202207301][PMID: 36748276]

[46] Kiefer AF, Schütz C, Englisch CN, *et al.* Dipeptidic Phosphonates: Potent Inhibitors of Pseudomonas aeruginosa Elastase B Showing Efficacy in a Murine Keratitis Model. Adv Sci (Weinh) 2025: e2411807 [https://doi.org/10.1002/advs.202411807][PMID: 39973061]

[68] Aljohmani A, Opitz B, Bischoff M, Yildiz D. Pseudomonas aeruginosa Triggered Exosomal Release of ADAM10 Mediates Proteolytic Cleavage in Trans. Int J Mol Sci 2022; 23(3) [https://doi.org/10.3390/ijms23031259][PMID: 35163191]

[69] Edgar R, Domrachev M, Lash AE. Gene expression omnibus: NCBI gene expression and hybridization array data repository. Nucleic Acids Res. 2002; 30(1):207-210. [https://doi.org/10.1093/nar/30.1.207] [PMID: 11752295]
