# Supplementary material for: Exploring the Role of Pseudomonas aeruginosa Elastase in Lung Epithelial Barrier Dysfunction: Advancing toward Antivirulence Therapies
Source: ACS Infect Dis. 2026 Apr 10;12(5):1611–26. doi: 10.1021/acsinfecdis.5c00915 (PMC13162270; doi:10.1021/acsinfecdis.5c00915)
Supplement: Supplementary file 2 [file id5c00915_si_002.zip › Calu-3 LASB Pathway Analysis Reactome.pdf]

Reactome Analysis Result

05 June, 2025

Introduction

This report summarizes the pathway analysis result created by the **Reactome Analysis Service**. For more information, visit https://www.reactome.org.

In this reports, pathways found differentially expressed at an adjusted p-value <= 0.05 are considered to be significantly regulated.

Analysis Overview

- Number of datasets analysed: 1
- Reactome version: 92
- Disease pathways were included

Dataset summaries

normalized__counts__table modify reactome:

- 2692 pathways
- 9540 fold changes for genes / proteins

Pathway analysis

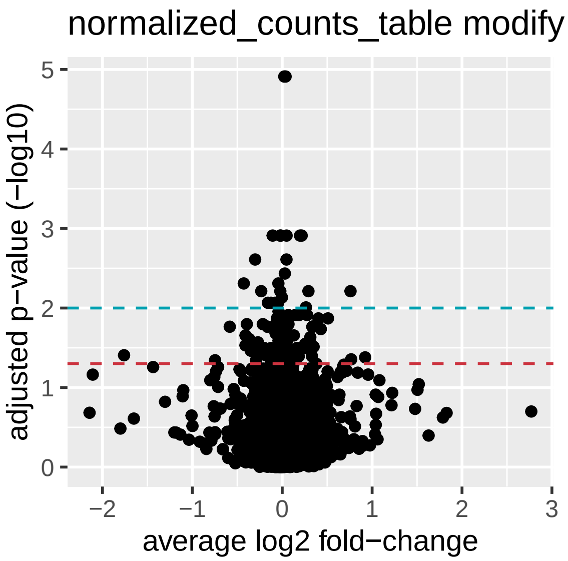

These volcano plots summarize the pathway results for every dataset. Every point is one pathway. The x-axis represents the average fold-change of all genes / proteins within that pathway. The y-axis represents the p-value where "higher" values are more significant (-log10 transformation). The red line represents p = 0.05, and the blue line p = 0.01.

Note
Since this result only contains a single datasets, some analyses are not included in this report.

Top-regulated pathways

Cell-cell junction organization (R-HSA-421270)

	normalized_counts_table modify reactome
Regulation	**Up**
FDR	**< 0.001**
Av. FC	0.037
N Genes	77

NrCAM interactions (R-HSA-447038)

	normalized_counts_table modify reactome
Regulation	**Up**
FDR	**< 0.001**
Av. FC	0.023
N Genes	7

Breakdown of the nuclear lamina (R-HSA-352238)

	normalized_counts_table modify reactome
Regulation	**Up**
FDR	**0.001**
Av. FC	0.198
N Genes	3

RHO GTPases Activate Rhotekin and Rhophilins (R-HSA-5666185)

	normalized_counts_table modify reactome
Regulation	**Up**
FDR	**0.001**
Av. FC	0.216
N Genes	9

Trafficking of myristoylated proteins to the cilium (R-HSA-5624138)

	normalized_counts_table modify reactome
Regulation	**Up**
FDR	**0.001**
Av. FC	0.048
N Genes	5

Cell junction organization (R-HSA-446728)

	normalized_counts_table modify reactome
Regulation	**Up**
FDR	**0.002**
Av. FC	0.047
N Genes	104

Developmental Biology (R-HSA-1266738)

	normalized_counts_table modify reactome
Regulation	**Up**
FDR	**0.004**
Av. FC	0.029
N Genes	1181

OGDH complex synthesizes succinyl-CoA from 2-OG (R-HSA-9853506)

	normalized_counts_table modify reactome
Regulation	**Up**
FDR	**0.006**
Av. FC	0.291
N Genes	4

Defective MMAB causes MMA, cblB type (R-HSA-3359471)

	normalized_counts_table modify reactome
Regulation	**Up**
FDR	**0.006**
Av. FC	0.76
N Genes	1

Negative regulation of MAPK pathway (R-HSA-5675221)

	normalized_counts_table modify reactome
Regulation	**Up**
FDR	**0.01**
Av. FC	0.264
N Genes	42

Acyl chain remodeling of CL (R-HSA-1482798)

	normalized_counts_table modify reactome
Regulation	**Up**
FDR	**0.011**
Av. FC	0.246
N Genes	6

Defective visual phototransduction due to LRAT loss of function (R-HSA-9918442)

	normalized_counts_table modify reactome
Regulation	**Up**
FDR	**0.012**
Av. FC	0.186
N Genes	2

PLCG1 events in ERBB2 signaling (R-HSA-1251932)

	normalized_counts_table modify reactome
Regulation	**Up**
FDR	**0.012**
Av. FC	0.277
N Genes	4

PTK6 promotes HIF1A stabilization (R-HSA-8857538)

	normalized_counts_table modify reactome
Regulation	**Up**
FDR	**0.012**
Av. FC	0.068
N Genes	6

TFAP2 (AP-2) family regulates transcription of cell cycle factors (R-HSA-8866911)

	normalized_counts_table modify reactome
Regulation	**Up**
FDR	**0.012**
Av. FC	0.143
N Genes	5

RUNX2 regulates bone development (R-HSA-8941326)

	normalized_counts_table modify reactome
Regulation	**Up**
FDR	**0.013**
Av. FC	0.014
N Genes	29

Deletions in the AXIN1 gene destabilize the destruction complex (R-HSA-5467345)

	normalized_counts_table modify reactome
Regulation	**Up**
FDR	**0.013**
Av. FC	0.509
N Genes	1

Linoleic acid (LA) metabolism (R-HSA-2046105)

	normalized_counts_table modify reactome
Regulation	**Up**
FDR	**0.013**
Av. FC	0.403
N Genes	8

Adherens junctions interactions (R-HSA-418990)

	normalized_counts_table modify reactome
Regulation	**Up**
FDR	**0.016**
Av. FC	0.07
N Genes	47

NR1H2 and NR1H3-mediated signaling (R-HSA-9024446)

	normalized_counts_table modify reactome
Regulation	**Up**
FDR	**0.017**
Av. FC	0.026
N Genes	45

LRR FLII-interacting protein 1 (LRRFIP1) activates type I IFN production (R-HSA-3134973)

	normalized_counts_table modify reactome
Regulation	**Down**
FDR	**0.001**
Av. FC	-0.023
N Genes	5

Oncogenic MAPK signaling (R-HSA-6802957)

	normalized_counts_table modify reactome
Regulation	**Down**
FDR	**0.001**
Av. FC	-0.016
N Genes	82

Signaling by BRAF and RAF1 fusions (R-HSA-6802952)

	normalized_counts_table modify reactome
Regulation	**Down**
FDR	**0.001**
Av. FC	-0.107
N Genes	65

Uptake and function of anthrax toxins (R-HSA-5210891)

	normalized_counts_table modify reactome
Regulation	**Down**
FDR	**0.002**
Av. FC	-0.301
N Genes	11

TRAF6 mediated IRF7 activation (R-HSA-933541)

	normalized_counts_table modify reactome
Regulation	**Down**
FDR	**0.005**
Av. FC	-0.043
N Genes	19

Activation of BIM and translocation to mitochondria (R-HSA-111446)

	normalized_counts_table modify reactome
Regulation	**Down**
FDR	**0.005**
Av. FC	-0.428
N Genes	3

Regulation of MITF-M dependent genes involved in metabolism (R-HSA-9854907)

	normalized_counts_table modify reactome
Regulation	**Down**
FDR	**0.006**
Av. FC	-0.234
N Genes	4

Transcriptional regulation by RUNX2 (R-HSA-8878166)

	normalized_counts_table modify reactome
Regulation	**Down**
FDR	**0.006**
Av. FC	-0.022
N Genes	102

Cell-Cell communication (R-HSA-1500931)

	normalized_counts_table modify reactome
Regulation	**Down**
FDR	**0.007**
Av. FC	-0.004
N Genes	139

Regulation of gene expression by Hypoxia-inducible Factor (R-HSA-1234158)

	normalized_counts_table modify reactome
Regulation	**Down**
FDR	**0.009**
Av. FC	-0.126
N Genes	10

TP53 regulates transcription of additional cell cycle genes whose exact role in the p53 pathway remain uncertain (R-HSA-6804115)

	normalized_counts_table modify reactome
Regulation	**Down**
FDR	**0.009**
Av. FC	-0.159
N Genes	21

TFAP2 (AP-2) family regulates transcription of growth factors and their receptors (R-HSA-8866910)

	normalized_counts_table modify reactome
Regulation	**Down**
FDR	**0.009**
Av. FC	-0.045
N Genes	13

RHOB GTPase cycle (R-HSA-9013026)

	normalized_counts_table modify reactome
Regulation	**Down**
FDR	**0.009**
Av. FC	-0.08
N Genes	69

Tight junction interactions (R-HSA-420029)

	normalized_counts_table modify reactome
Regulation	**Down**
FDR	**0.011**
Av. FC	-0.041
N Genes	28

Lysosome Vesicle Biogenesis (R-HSA-432720)

	normalized_counts_table modify reactome
Regulation	**Down**
FDR	**0.012**
Av. FC	-0.031
N Genes	34

Downregulation of SMAD2/3:SMAD4 transcriptional activity (R-HSA-2173795)

	normalized_counts_table modify reactome
Regulation	**Down**
FDR	**0.013**
Av. FC	-0.058
N Genes	30

RND2 GTPase cycle (R-HSA-9696270)

	normalized_counts_table modify reactome
Regulation	**Down**
FDR	**0.016**
Av. FC	-0.063
N Genes	43

Signaling by TGFB family members (R-HSA-9006936)

	normalized_counts_table modify reactome
Regulation	**Down**
FDR	**0.016**
Av. FC	-0.032
N Genes	148

SUMOylation of transcription factors (R-HSA-3232118)

	normalized_counts_table modify reactome
Regulation	**Down**
FDR	**0.016**
Av. FC	-0.216
N Genes	20

Regulation of MITF-M-dependent genes involved in apoptosis (R-HSA-9824594)

	normalized_counts_table modify reactome
Regulation	**Down**
FDR	**0.016**
Av. FC	-0.394
N Genes	17

Protein / Gene level results

Classical differential expression analysis performed on the genes / proteins of every dataset.

Note: Depending on the gene set analysis method used, the approach used to assess differential expression at the gene / protein level may vary to the approach used for the pathway level.

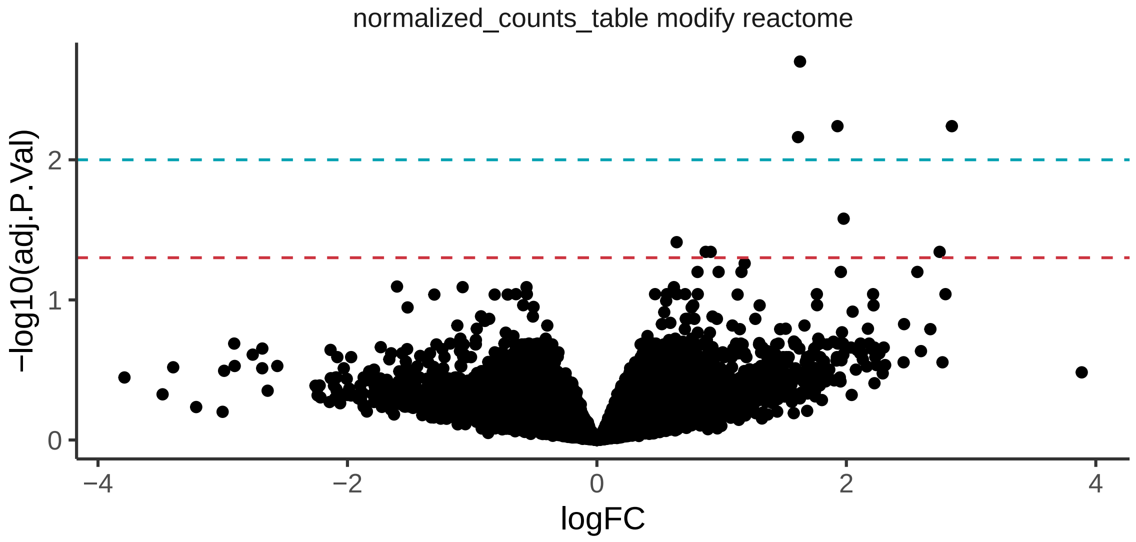

Top up-regulated protein/genes

normalized__counts__table modify reactome

Identifier	logFC	AveExpr	t	P.Value	adj.P.Val	B
ENSG00000151726	1.6284963	5.950999	7.739734	2.00e-07	0.0019899	7.280308
ENSG00000158050	2.8459353	2.419175	6.671083	1.80e-06	0.0057529	5.136127
ENSG00000134955	1.9287146	1.183061	6.788547	1.40e-06	0.0057529	5.048087
ENSG00000086696	1.6124715	4.409303	6.449096	2.90e-06	0.0068888	4.779562
ENSG00000114812	1.9783323	4.455945	5.727705	1.38e-05	0.0263253	3.305547
ENSG00000075142	0.6393239	5.822152	5.472307	2.44e-05	0.0387309	2.758117
ENSG00000137440	2.7478761	3.821025	5.245901	4.06e-05	0.0454095	2.286615
ENSG00000139211	0.8720985	4.480779	5.221657	4.28e-05	0.0454095	2.237928
ENSG00000141736	0.9125061	14.643373	5.244988	4.06e-05	0.0454095	2.234701
ENSG00000185813	1.1848139	4.791334	5.091761	5.75e-05	0.0548888	1.949990

Top down-regulated protein/genes

normalized__counts__table modify reactome

Identifier	logFC	AveExpr	t	P.Value	adj.P.Val	B
ENSG00000005108	-1.6025758	2.1096980	-4.720804	0.0001346	0.0802601	0.9879954
ENSG00000164105	-1.0764474	3.4352651	-4.668276	0.0001519	0.0810774	1.0479646
ENSG00000131941	-0.5643185	7.9094896	-4.641921	0.0001615	0.0810774	0.9269234

Identifier	logFC	AveExpr	t	P.Value	adj.P.Val	B
ENSG00000157933	-0.5614794	6.8124616	-4.543745	0.0002026	0.0910062	0.7266451
ENSG00000148175	-0.6490260	6.9466426	-4.409376	0.0002766	0.0910062	0.4270469
ENSG00000187098	-0.8194873	6.1176348	-4.374375	0.0003001	0.0916048	0.3783607
ENSG00000105939	-0.7153398	6.6376625	-4.366789	0.0003054	0.0916048	0.3416612
ENSG00000112782	-1.3033676	0.0588558	-4.363917	0.0003074	0.0916048	-0.0746288
ENSG00000107036	-0.5908355	5.2888653	-4.240544	0.0004095	0.1090889	0.1126807
ENSG00000171150	-0.5078578	5.4407748	-4.180691	0.0004706	0.1122479	-0.0285667
